# Susceptibility to infection with *Borrelia afzelii* and TLR2 polymorphism in a wild reservoir host

**DOI:** 10.1038/s41598-019-43160-3

**Published:** 2019-04-30

**Authors:** Andrea Gomez-Chamorro, Florian Battilotti, Claire Cayol, Tapio Mappes, Esa Koskela, Nathalie Boulanger, Dolores Genné, Anouk Sarr, Maarten Jeroen Voordouw

**Affiliations:** 1 Institut de Biologie, Université de Neuchâtel, Neuchâtel, Switzerland; 2 Department of Biological and Environmental Science, University of Jyväskylä, Jyväskylä, Finland; 3 Facultés de Médecine et de Pharmacie, Université de Strasbourg, Strasbourg, France; 4 Department of Veterinary Microbiology, Western College of Veterinary Medicine, University of Saskatchewan, Saskatoon, Canada

**Keywords:** Ecological genetics, Immunogenetics

## Abstract

The study of polymorphic immune genes in host populations is critical for understanding genetic variation in susceptibility to pathogens. Controlled infection experiments are necessary to separate variation in the probability of exposure from genetic variation in susceptibility to infection, but such experiments are rare for wild vertebrate reservoir hosts and their zoonotic pathogens. The bank vole (*Myodes glareolus*) is an important reservoir host of *Borrelia afzelii*, a tick-borne spirochete that causes Lyme disease. Bank vole populations are polymorphic for Toll-like receptor 2 (TLR2), an innate immune receptor that recognizes bacterial lipoproteins. To test whether the TLR2 polymorphism influences variation in the susceptibility to infection with *B*. *afzelii*, we challenged pathogen-free, lab-born individuals of known TLR2 genotype with *B*. *afzelii*-infected ticks. We measured the spirochete load in tissues of the bank voles. The susceptibility to infection with *B*. *afzelii* following an infected tick bite was very high (95%) and did not differ between TLR2 genotypes. The TLR2 polymorphism also had no effect on the spirochete abundance in the tissues of the bank voles. Under the laboratory conditions of our study, we did not find that the TLR2 polymorphism in bank voles influenced variation in the susceptibility to *B*. *afzelii* infection.

## Introduction

The Toll-like receptors (TLRs) are an important family of pathogen recognition receptors (PRRs) that are found in all vertebrates^1^. These receptors of the innate immune system play a critical role in the recognition of pathogen-associated molecular patterns (PAMPs). PAMPs are highly conserved molecules that are essential for the pathogen’s existence such as the cell wall components of bacteria. To date, 12 to 15 different TLR genes have been discovered in humans and mice, and each TLR receptor recognizes a distinct set of PAMPs^2^. For example, TLR4 recognizes lipopolysaccharides (LPS), whereas TLR5 recognizes the bacterial flagellin protein^2,3^. The recognition of pathogens by the TLR receptors is a critical step in the activation of the adaptive immune system in the vertebrate host^4^. Recent reviews have described numerous polymorphisms in the different TLRs and their associations with susceptibility or resistance to different infectious diseases^4,5^.

The toll-like receptor 2 (TLR2) recognizes a wide variety of PAMPs including peptidoglycan, zymosan, and lipoproteins^3^. TLR2 can also dimerize with TLR1 and TLR6 to recognize additional PAMPs^6^. TLR2 plays an important role in recognizing the lipoproteins of the spirochete bacteria of the *Borrelia burgdorferi* sensu lato (sl) genospecies complex, which includes the causative agents of Lyme borreliosis in humans^7,8^. Functional studies have shown that TLR2-knockout mice have much higher loads of *B*. *burgdorferi* sensu stricto (ss) in their tissues than immunocompetent control mice^7,9^. Furthermore, population genetic studies have found associations between genetic polymorphisms at the TLR2 locus and resistance to *B*. *burgdorferi* sl pathogens in humans and wild rodents^10–14^.

Lyme borreliosis is the most common vector-borne disease in the northern hemisphere^15^. In Europe, *Borrelia afzelii* is the most important etiological agent of Lyme borreliosis and is transmitted by the hard tick *Ixodes ricinus*^16,17^. The main reservoir hosts of *B*. *afzelii* include small mammals such as mice (*Apodemus* species) and the bank vole (*Myodes glareolus*). Experimental infections have shown that *B*. *afzelii* establishes chronic infections in its rodent reservoir hosts that can last for months or even years^18–20^. Larval ticks acquire spirochetes after feeding on an infected reservoir host, as there is no vertical transmission^21,22^. Blood-engorged larvae subsequently moult into nymphs that infect the reservoir hosts the following year^15^. In areas with high tick densities, wild rodents are repeatedly bitten by nymphs and exposed to *B*. *burgdorferi* sl pathogens^23,24^. Long-term field studies in Sweden have shown that a quarter of all wild rodents were infected with *B*. *afzelii*^25^. Thus, *B*. *afzelii* is an important pathogen in wild rodent populations that could exert strong selection on the host immune system^13,14,26^.

The bank vole (*M*. *glareolus*) is an important wild reservoir host for *B*. *afzelii* and for immature *Ixodes* ticks^17,23,27^. Genetic studies on the TLR2 locus in this species found three different clusters of alleles: C1, C2, and C3^13,28^. A field study in Sweden found that individuals with the C1C1, C1C2, and C2C2 genotype had the highest (48.6%), intermediate (30.6%), and lowest (17.9%) prevalence of *B*. *afzelii*^13^. Thus, the C1 and C2 alleles appear to confer susceptibility and resistance, respectively, to *B*. *afzelii* in bank voles. The C3 allele was rare in this population and its phenotype with respect to *B*. *afzelii* infection was not investigated. A recent study of the TLR2 polymorphism in bank vole populations across 19 countries in Europe found a strong positive correlation between the frequency of the C2 resistance allele and the case load of human Lyme borreliosis^26^. This study suggests that *B*. *afzelii* is driving the evolution of the C2 resistance allele in European bank vole populations^26^. Nevertheless, it remains to be conclusively demonstrated that the TLR2 polymorphism in bank voles causes variation in susceptibility to *B*. *afzelii* in this important reservoir host.

To date, all the work on the TLR2 polymorphism and *B*. *afzelii* infection in bank voles has been correlative in nature^13,14,26^. Experimental infections are needed to further confirm whether the TLR2 polymorphism in this species influences variation in susceptibility to infection with *B*. *afzelii*. The two aims of this study were to determine whether the TLR2 polymorphism in wild bank voles influences variation in the probability of infection with *B*. *afzelii* (hereafter referred to as host susceptibility) and variation in *B*. *afzelii* spirochete abundance in host tissues (hereafter referred to as host spirochete load). To test this hypothesis, we challenged *Borrelia*-free, lab-born bank voles of known TLR2 genotype with *B*. *afzelii* via tick bite. The TLR2 gene could influence the prevalence of *B*. *afzelii* by different mechanisms such as preventing systemic infection and/or spirochete clearance following infection. We expected that compared to the C1C1 genotype, the C2C2 genotype would be less likely to develop a systemic infection following infestation with *B*. *afzelii*-infected nymphs. We also expected that the C2C2 genotype would have a lower spirochete load in the tissues than the C1C1 genotype. The importance of the C3 allele was assessed for the first time in this study.

## Materials and Methods

### General experimental design

The present study consists of two separate infection experiments: the first experiment was conducted in the summer of 2015 using bank voles from a Swiss colony, whereas the second experiment was conducted in the summer of 2016 using bank voles from a Finnish colony. In these infection experiments, the Swiss and Finnish bank voles of known TLR2 genotype were challenged via tick bite with different isolates of *B*. *afzelii*, respectively. The Swiss bank vole colony contained the C1 susceptible allele and the C3 allele, for which the *B*. *afzelii* infection phenotype was unknown. The Finnish bank vole colony contained the C1 susceptible allele, the C2 resistance allele, and the C3 allele.

### Ethics statement and animal experimentation permits

All the experiments were performed following the Swiss and Finnish legislation on animal experimentation. For the infection experiment conducted in Switzerland, the commission that is part of the ‘Service de la Consommation et des Affaires Vétérinaires (SCAV)’ of the canton of Vaud, Switzerland evaluated and approved the ethics of this part of the study. The SCAV of the canton of Neuchâtel, Switzerland issued the animal experimentation permit (NE2/2014). The infection experiment conducted in Finland was carried out under the permits of the Finnish Animal Experiment Board (ESAVI/3834/04.10.03/2011, ESAVI/7256/04.10.07/2014 and ESAVI/3457/04.10.07/2015).

### Breeding of the bank voles in the laboratory

The Swiss and Finnish colonies of bank voles were established by trapping bank voles near Neuchâtel, Switzerland and Jyväskylä, Finland, respectively (section 1 in the electronic supplementary material (ESM)). TLR2 genotyping found that the Swiss bank voles carried the C1 and C3 alleles (n = 36 animals; frequencies were 42.2% and and 57.8%, respectively), but not the C2 putative resistance allele (section 2 in the ESM). For the Swiss infection experiment, male and female bank voles of known TLR2 genotype were crossed to maximize the number of homozygous C1C1 and C3C3 offspring. A sample of the Finnish colony (n = 48 animals) revealed that the frequencies of the TLR2 allele were as follows: 88.5% C1, 4.2% C2, and 7.3% C3. For the Finnish infection experiment, bank voles were bred to maximize the number of homozygous C1C1, C2C2 and C3C3 offspring. All lab-born offspring were genotyped with respect to their TLR2 locus. TLR2 genotyping of the bank voles is described in section 2 in the ESM.

### Strains of B. afzelii

Our original intention was to challenge the Swiss and Finnish bank voles with ticks carrying a single Swiss strain (NE4049) and a single Finnish strain (Fin-Jyv-A3) of *B*. *afzelii*, respectively. We chose these two strains because our previous work had shown that they are competent at establishing infection in laboratory mice^29–32^. Due to a combination of experimental error and time constraints, we used nymphs that were co-infected with two strains of *B*. *afzelii*. The Swiss bank voles were challenged with nymphs co-infected with a local Swiss strain (NE4049) and a foreign Austrian strain (E61). The Finnish bank voles were challenged with nymphs that were co-infected with a local Finnish strain (Fin-Jyv-A3) and a foreign Swiss strain (NE4049). The origin and genetics of these *B*. *afzelii* strains is described in section 3 in the ESM. We used a strain-specific qPCR to confirm that the local strain of *B*. *afzelii* had established infection in the bank voles (section 3 in the ESM).

### Infectious challenge of bank voles with *B*. *afzelii*-infected nymphal ticks

All animals were *Borrelia*-free, lab-born individuals and were in the adult stage (older than 2 months) at the time of the challenge with *B*. *afzelii*-infected nymphs. For the Swiss infection experiment, 50 animals were challenged with *B*. *afzelii*-infected nymphs and the TLR2 genotypes were as follows: 17 C1C1 (12 females, 5 males), 15 C1C3 (7 females, 8 males), and 18 C3C3 (8 females, 10 males). For the Finnish infection experiment, 50 animals were challenged and their TLR2 genotypes were as follows: 11 C1C1 (4 females, 7 males), 8 C1C2 (4 females, 4 males), 12 C2C2 (4 females, 8 males), 1 C2C3 (1 male), 8 C1C3 (4 females, 4 males), and 10 C3C3 (6 females, 4 males).

In the field, rodents are rarely infested with more than one *I*. *ricinus* nymph at a time^13,23,24,33^. To simulate the natural conditions while also ensuring a good probability of infectious challenge, each animal was infested with 3 randomly selected nymphs. The creation of the experimentally infected nymphs is described in section 3 in the ESM. The percentage of *B*. *afzelii*-infected nymphs was >90% for both the Swiss and Finnish infection experiments. The nymphs were placed in a neoprene capsule that had been glued to the shaved backs of the bank voles. During this procedure, animals were anesthetized with a mixture of xylazine, ketamine and PBS (1:2:9; 5 µl per gram of bank vole). The purpose of the capsule is to prevent the bank vole from grooming off and killing the attached nymphs. The animals were fitted with an Elizabethan collar to prevent them from removing the capsules. The capsules were checked on a daily basis over a period of 7 days, and detached engorged nymphs were collected from the capsule and frozen at −20 °C.

At 49 days post-infection, the animals were sacrificed using CO_2_ asphyxiation. Our previous work had shown that this is enough time for *B*. *afzelii* to disseminate to all of the organs^20,29,30^. For each animal, a tissue biopsy was taken from the ear and the dorsal skin from the site of the tick bite using a 2 mm forceps-type punch. The bladder and ankle joints were aseptically dissected^29^. These internal tissues are typically used to demonstrate that *B*. *burgdorferi* sl has disseminated and established a systemic infection^9,34–38^. To determine the infection status of the bank voles, these four tissue samples were tested for *B*. *afzelii* using qPCR (see below).

### *Borrelia afzelii* infection status of the engorged nymphs and the bank voles

We extracted the DNA from engorged nymphs and bank vole tissue samples and used qPCR targeting the *flagellin* gene to estimate the spirochete load (for details, see section 3 in the ESM). For the bank vole tissue samples, we used the DNA concentration of the DNA extraction to standardize the spirochete load as the number of spirochetes per mg of host DNA (for details, see section 3 in the ESM).

### Statistical analyses

#### General approach

The three response variables included the number of engorged nymphs collected from each bank vole, the infection status of the bank vole (infected, uninfected) and the spirochete load of *B*. *afzelii* in the bank vole tissues. These three response variables were analysed using generalized linear models (GLMs) with binomial errors, GLMs with binomial errors, and linear mixed effects models (LMEMs) with normal errors, respectively. The fixed effects included experiment (2 levels: Swiss, Finnish), sex (2 levels: female, male), organ (4 levels: bladder, ear, joint, dorsal skin), and TLR2 genotype. TLR2 genotype was coded in eight different ways that corresponded to different assumptions about the effects of the TLR2 alleles (Table 1).

**Table 1:** The eight different ways in which TLR2 genotype was modelled are shown.

Name	Type of variable	# of genotypes	Identity of genotypes	# of alleles	Identity of alleles
Geno1	Categorical	6	C1C1, C2C2, C3C3, C1C2, C1C3, C2C3	NA	NA
Geno2	Categorical	3	C2C2, C2Cx, CxCx; (Cx = C1 = C3)	NA	NA
Geno3	Categorical	3	C1C1, C1Cy, CyCy; (Cy = C2 = C3)	NA	NA
Geno4	Categorical	3	C3C3, C2Cz, CzCz; (Cz = C1 = C2)	NA	NA
Geno5	1 Covariate	NA	NA	2	# of C2 alleles; (C1 = C3)
Geno6	1 Covariate	NA	NA	2	# of C1 alleles; (C2 = C3)
Geno7	1 Covariate	NA	NA	2	# of C3 alleles; (C1 = C2)
Geno8	3 Covariates	NA	NA	3	# of C1, C2, and C3 alleles

TLR2 genotype was either modelled as a categorical factor where each TLR2 genotype was a different category or as a covariate that counted the number of TLR2 alleles. We reduced the number of genotypes or alleles by setting pairs of alleles or genotypes as equivalent. For example, for Geno2, we assumed that the C1 and C3 alleles are equivalent so that there are only 3 distinct genotypes: C2C2, C2Cx, CxCx, where Cx = C1 = C3. Combining similar genotypes (or alleles) increases the sample size for the remaining genotype categories and thereby increases the power of the statistical test.

Experiment and TLR2 could not be included in the same model because of partial or complete redundancy between these factors (i.e. the C2 allele only occurs in the Finnish experiment). We therefore used model selection based on the Akaike Information Criterion (AIC) to determine the best model; the advantage of this approach is that it allows us to compare non-nested models. We used the model weights to calculate the % support for each of the fixed effects. We used model averaging to obtain robust parameter estimates and 95% confidence intervals (95% CI) for the fixed effects. A fixed effect is significant when its model-averaged 95% CI does not overlap 0. We used R version 3.4.3 to analyse the data. The GLMs and LMEMs were run using the glm() function in the base package and the lmer() function in the lme4 package, respectively. Model selection and calculation of model-averaged parameter estimates were run using the model.sel() and the model.avg() functions in the MuMIn package.

#### Number of engorged nymphs per bank vole

Each bank vole was infested with 3 nymphs, and the number of engorged nymphs collected from each bank vole was therefore a binomial response variable that ranged from 0/3 to 3/3. To test whether the 100 bank voles were exposed to the same infectious challenge, we analysed two response variables: the number of engorged nymphs that were collected per bank vole and the number of engorged *B*. *afzelii*-infected nymphs that were collected per bank vole. These two response variables were modelled using GLMs with binomial errors. The fixed factors were experiment, sex, and the eight different ways to code TLR2 genotype. Each vole occurred only once in the analysis so it was not necessary to include bank vole ID as a random effect.

#### TLR2 genotype and infection status of the bank voles

Bank vole infection status is a binomial variable (uninfected, infected) that was modelled using GLMs with binomial errors. The fixed effects were number of *B*. *afzelii*-infected nymphs (0, 1, 2, 3), experiment, sex, and the eight different ways to code TLR2 genotype. As there was no variation in infection status among the four organs, we did not include this factor in the analysis. Each bank vole occurred only once in the analysis so it was not necessary to include bank vole ID as a random effect. The analysis was performed for all the bank voles (n = 100) and for the subset of bank voles that had been successfully challenged with *B*. *afzelii*-infected nymphs (n = 88).

#### *B*. *afzelii* spirochete load of the bank vole tissue samples

The statistical analysis of the *B*. *afzelii* spirochete load in the tissue samples was restricted to the subset of infected bank voles (n = 84). For each tissue sample, the geometric mean spirochete load (in 3 μl of DNA template) was calculated for the three replicate qPCR runs. The tissue spirochete loads were standardized to mg of DNA (by dividing by the DNA concentration). This variable was log10-transformed to normalize the residuals. The log10-transformed tissue spirochete loads were modelled using LMEMs with normal errors. The fixed factors were experiment, sex, organ, and the eight different ways to code TLR2 genotype. Organs sampled from the same bank vole are not independent and bank vole identity was therefore modelled as a random factor.

## Results

### The C1, C2, and C3 allele clusters encode different TLR2 proteins

The TLR2 gene sequences of the animals in the experimental infection formed three clearly separated clusters that coded for three different protein variants: cluster 1 (C1), cluster 2 (C2) and cluster 3 (C3); these three clusters were the same as the ones described by Tschirren *et al*.^13^. The C1 and C2 clusters, C1 and C3 clusters, and C2 and C3 clusters were separated by a genetic distance of 12, 15, and 15 nucleotide differences, respectively, which corresponded to a protein distance of 7, 7, and 4 amino acids, respectively (Fig. S1). A protein model of TLR2 had previously shown that the variants encoded by the C1 and C2 alleles would differ in at least one amino acid in the binding site that is believed to be critical for ligand binding^13^. We therefore expected that these three variants of the TLR2 protein would differ in their ability to recognize the lipoproteins of *B*. *afzelii* and therefore to protect the bank vole against infection via tick bite.

### Definition of a successful infectious challenge

We collected at least 1 engorged *B*. *afzelii*-infected nymph from 83 of the 100 bank voles (Tables S01 and S02 in the ESM). There were 17 bank voles for which we did not recover any engorged *B*. *afzelii*-infected nymphs (Tables S01 and S02 in the ESM). Five of these 17 individuals became infected with *B*. *afzelii* proving that they had been exposed to an infected nymph (which we failed to recover). Hence, we have proof that 88 of the 100 bank voles were successfully challenged in this study (83 + 5 = 88). The 12 remaining individuals for which there was no proof of an infectious challenge (no infected engorged nymphs, bank voles are uninfected) all came from the Swiss experiment.

### Number of engorged nymphs per bank vole

For the 50 Swiss bank voles, we collected 84 engorged nymphs (mean = 2.00, range = 0–3 engorged nymphs per bank vole), of which 52 were infected with *B*. *afzelii* (mean = 1.24, range = 0–3 engorged infected nymphs per bank vole). For the 50 Finnish bank voles, we collected 131 engorged nymphs (mean = 2.72, range = 2–3 engorged nymphs per bank vole), of which 96 were infected with *B*. *afzelii* (mean = 1.92, range = 0–3 engorged infected nymphs per bank vole).

For the model selection analysis of the number of engorged nymphs per bank vole, the top 3 models had 99.999% of the support; the remaining 26 models had <0.001% of the support (Table 2 and Table S07 in the ESM). The support for individual factors was as follows: experiment (99.999%), sex (32.4%), and TLR2 genotype (<0.001% for 24 models). The model-averaged parameter estimates found that significantly more engorged nymphs were collected per bank vole in the Finnish experiment (Table S08 in the ESM) compared to the Swiss experiment.

**Table 2:** Model selection table is shown for the generalized linear models (with binomial errors) of the number of engorged nymphs per bank vole (nymphs.engorged).

Model	Model structure	df	logLik	AICc	Delta	Weight (%)
model003	nymphs.engorged~E	2	−109.353	222.831	0.000	67.575
model002	nymphs.engorged~E + S	3	−109.316	224.881	2.051	24.235
model001	nymphs.engorged~E + S + E:S	4	−109.315	227.051	4.221	8.189
model010	nymphs.engorged~geno2	3	−121.250	248.751	25.920	0.000
model022	nymphs.engorged~geno5	2	−122.638	249.399	26.569	0.000

The analysis was done on the entire data set of 100 bank voles. Of the 29 models, the top 5 models are shown; the top 3 models have 99.999% of the support. Table S09 in the ESM shows all 29 models. The fixed factors are experiment (E), sex (S), and TLR2 genotype. TLR2 genotype was modelled in 8 different ways (geno1, geno2, geno3, geno4, geno5, geno6, geno7, and geno8). For each model, the model structure, model degrees of freedom (df), log likelihood, corrected AIC value (AICc), difference in AICc from the top model (Delta), and the support (Weight) expressed as a percent are shown.

The results were similar for the number of engorged *B*. *afzelii*-infected nymphs and when the analyses were done on the subset of 88 bank voles that had been successfully challenged. These analyses show that the infectious challenge was the same for all TLR2 genotypes.

### Infection status of the bank voles

A bank vole was defined as being infected if it tested positive for *B*. *afzelii* for at least one of four organs: (1) bladder, (2) ear, (3) joint, and (4) dorsal skin. There was no ambiguity about the infection status of the 100 bank voles: 84 tested positive for all four organs and 16 tested negative for all four organs. The susceptibility of a host to a pathogen is the probability of acquiring an infection following exposure. In our study, the susceptibility of bank voles to infection with *B*. *afzelii* was 95.5% (84 infected/ 88 total; 95% CI = 88.1–98.5%) and was therefore very high.

Infection was higher in the Finnish experiment (100.0% = 50/50) than the Swiss experiment (68.0% = 34/50). When the analysis was restricted to the subset of 88 bank voles that had been successfully challenged, infection in the Finnish experiment (100.0% = 50/50) and Swiss experiment (89.5% = 34/38) were similar.

Bank vole infection status was initially modelled using GLMs with binomial errors (Table S09 in the ESM). However, due to the lack of variation in infection status (most bank voles were infected), the standard errors (SE) estimated by the glm() function had very low precision (i.e., SE was ~1000 times bigger than the parameter estimate; Table S10 in the ESM). To avoid this problem with parameter estimation, we re-analysed the data using linear models with normal errors.

For the model selection analysis of the linear models of infection status of 100 bank voles, the top 6 models had 99.9% of the support; the remaining 111 models had 0.1% of the support (Table 3 and Table S11 in the ESM). The support for individual factors was as follows: number of infected nymphs (>99.9%), experiment (>99.9%), sex (55.6%), and TLR2 genotype (<0.001% for 98 models). The model-averaged parameter estimates found that infection increased significantly with the number of engorged infected nymphs per bank vole and that infection was significantly higher in the Finnish experiment compared to the Swiss experiment (Table S12 in the ESM).

**Table 3:** Model selection table is shown for the linear models of bank vole infection status (infection).

Model	Model structure	df	logLik	AICc	Delta	Weight (%)
model011	infection~E + N + E:N	5	−12.287	35.212	0.000	44.374
model007	infection~E + S + N + E:N	6	−11.802	36.507	1.295	23.226
model005	infection~E + S + N + E:N + S:N	7	−11.164	37.546	2.334	13.817
model003	infection~E + S + N + E:S + E:N	7	−11.317	37.851	2.639	11.862
model002	infection ~E + S + N + E:S + E:N + S:N	8	−11.032	39.646	4.434	4.835
model001	infection~E + S + N + E:S + E:N + S:N + E:S:N	9	−10.826	41.651	6.439	1.774
model014	infection~E + N	4	−21.428	51.277	16.065	0.014
model606	infection~geno6 + S + N + geno6:S	6	−19.780	52.463	17.251	0.008
model009	infection ~E + X + N	5	−20.939	52.517	17.305	0.008
model008	infection ~E + X + N + S:N	6	−19.871	52.645	17.433	0.007

The analysis was done on the entire data set of 100 bank voles. Of the 117 models, the top 10 models are shown; the top 6 models have 99.9% of the support. Table S11 in the ESM shows all 117 models. The fixed factors are number of engorged *B*. *afzelii*-infected nymphs (N), experiment (E), sex (S), and TLR2 genotype. TLR2 genotype is modelled in 7 different ways (geno1, geno2, geno3, geno4, geno5, geno6, and geno7). For each model, the model structure, model degrees of freedom (df), log likelihood, corrected AIC value (AICc), difference in AICc from the top model (Delta), and the support (Weight) expressed as a percent are shown.

We do not present the model selection analysis for the subset of 88 bank voles that had been successfully challenged because there was no variation in infection status (84 infected/ 88 total).

**Figure 1:**
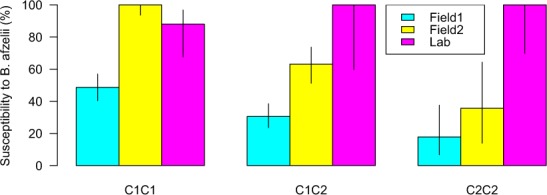
The susceptibility of the three bank vole TLR2 genotypes (C1C1, C1C2, and C2C2) to infection with *B*. *afzelii* is compared between our lab study (Lab) and the field study by Tschirren *et al*.^13^ (Field1, Field2). The field study by Tschirren *et al*.^13^ assumed that the exposure rates were identical for the three TLR2 genotypes and that bank voles do not clear the infection. Using these assumptions, the prevalence of infection can be separated into the probability of exposure and the susceptibility to infection following an infectious challenge. The Field1 estimates of susceptibility are based on the unrealistic assumption that 100% of the bank voles were exposed to an infected tick. The Field2 estimates of susceptibility are based on the assumption that 48.6% of the bank voles were exposed to an infected tick, which maximizes the susceptibility of the C1C1 genotype to 100.0% (for details, see section 4 in the ESM).

We compared the susceptibility of bank voles to infection with *B*. *afzelii* between our study and the study by Tschirren *et al*.^13^ (for details, see section 4 in the ESM). For the study by Tschirren *et al*.^13^, the probability of encountering an infected tick in the field was not known, but by setting this probability to a maximum (100%) or a minimum (48.7%), we were able to estimate the susceptibility of each TLR2 genotype. This comparison found that the susceptibility was significantly different between the two studies (Fig. 1).

### *B*. *afzelii* spirochete load of the bank vole tissue samples

**Figure 2:**
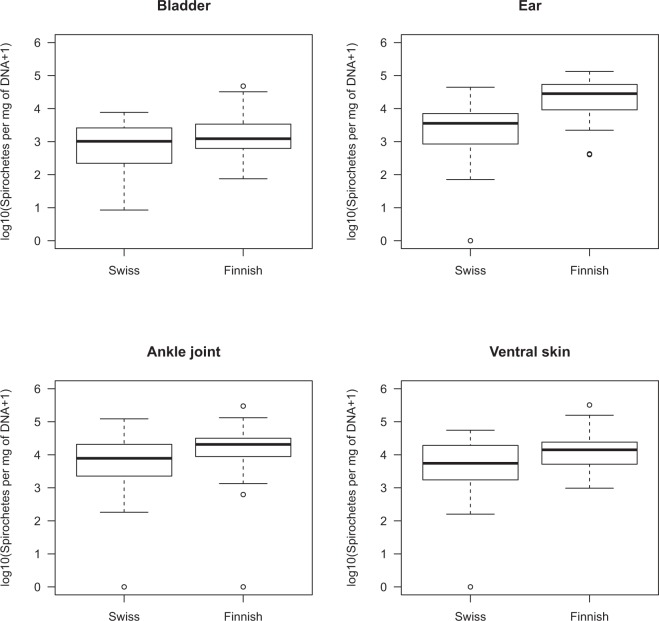
Organ and experiment had a significant effect on the tissue spirochete load of *B*. *afzelii* in the bank voles. The spirochete loads were standardized per mg of DNA and then log10-transformed. For the Finnish bank voles, the tissue spirochete loads were higher compared to the Swiss bank voles. The rank order of the organ spirochete loads (from lowest to highest) depended on the experiment: bladder, ear, ventral skin, ankle joint for the Swiss bank voles and bladder, ventral skin, ankle joint, ear for the Finnish bank voles. Shown are the medians (black line), the 25th and 75th percentiles (edges of the box), the minimum and maximum values (whiskers), and the outliers (circles).

For each organ, the repeatability of the log10-transformed tissue spirochete loads was high (range = 71–75%; section 5 in the ESM). For the Swiss bank voles, the mean spirochete load per mg of DNA was lowest in the bladder (540 spirochetes per mg of DNA; Table 4), 4.2 times higher in the ear, 5.1 times higher in the dorsal skin, and 8.4 times higher in the ankle joint (Fig. 2; Table 4). For the Finnish bank voles, the mean spirochete load per mg of DNA was lowest in the bladder (1339 spirochetes per mg of DNA; Table 4), 9.4 times higher in the dorsal skin, 10.9 times higher in the ankle joint, and 15.2 times higher in the ear (Fig. 2; Table 4). Averaged across the four organs, the tissue spirochete loads in the Finnish bank voles were 4.2 times higher than the Swiss bank voles (Fig. 2; Table 4).

**Table 4:** Mean tissue spirochete loads in the bank voles are shown for each organ in both the Swiss and Finnish infection experiments.

Experiment	Organ	Mean	LL	UL
Switzerland	Bladder	540	286	1017
Switzerland	Ear	2283	1211	4303
Switzerland	Joint	4531	2404	8541
Switzerland	Skin	2768	1469	5218
Finland	Bladder	1339	794	2259
Finland	Ear	20300	12036	34240
Finland	Joint	14533	8616	24512
Finland	Skin	12587	7463	21230

Bank vole organs were dissected at 49 days post-infection. The tissue spirochete load was standardized per mg of DNA (units are spirochetes per mg of DNA). Shown are the means, and the lower limits (LL) and upper limits (UL) of the 95% confidence interval.

**Table 5:** Model selection table is shown for the linear mixed effects models of the *B*. *afzelii* spirochete load in bank vole tissues (spiro.per.mg.DNA).

Model	Model structure	df	logLik	AICc	Delta	Weight (%)
lm.model1014	log10(spiro.per.mg.DNA)~E + O	7	−408.954	832.250	0.000	76.959
lm.model1011	log10(spiro.per.mg.DNA)~E + O + E:O	10	−407.676	836.029	3.779	11.632
lm.model1009	log10(spiro.per.mg.DNA)~E + S + O	8	−410.178	836.796	4.547	7.925
lm.model1006	log10(spiro.per.mg.DNA)~E + S + O + E:S	9	−410.639	839.830	7.580	1.739
lm.model1007	log10(spiro.per.mg.DNA)~E + S + O + E:O	11	−408.900	840.614	8.364	1.175
lm.model1003	log10(spiro.per.mg.DNA)~E + S + O + E:S + E:O	12	−409.361	843.688	11.438	0.253
lm.model1008	log10(spiro.per.mg.DNA)~E + S + O + S:O	11	−410.562	843.939	11.689	0.223
lm.model1004	log10(spiro.per.mg.DNA)~E + S + O + E:S + S:O	12	−411.023	847.012	14.762	0.048
lm.model1005	log10(spiro.per.mg.DNA)~E + S + O + E:O + S:O	14	−409.259	847.826	15.576	0.032
lm.model1002	log10(spiro.per.mg.DNA)~E + S + O + E:S + E:O + S:O	15	−409.720	850.939	18.690	0.007

Spirochete load is standardized per mg of DNA and was log10-transformed to normalize the residuals (log10(spiro.per.mg.DNA)). The analysis was done on the subset of 84 bank voles infected with *B*. *afzelii*. Of the 117 models, the top 10 models are shown; the top 5 models have 99.4% of the support. Table S12 in the ESM shows all 117 models. The fixed factors are experiment (E), organ (O), sex (S), and TLR2 genotype. TLR2 genotype is modelled in 7 different ways (geno1, geno2, geno3, geno4, geno5, geno6, and geno7). For each model, the model structure, model degrees of freedom (df), log likelihood, corrected AIC value (AICc), difference in AICc from the top model (Delta), and the support (Weight) expressed as a percent are shown.

For the model selection analysis of tissue spirochete load per mg of DNA, the top 5 models had 99.4% of the support; the remaining 112 models had 0.6% of the support (Table 5 and Table S13 in the ESM). The support for individual factors was as follows: experiment (99.99%), sex (11.4%), organ (100.0%), and TLR2 genotype (<0.001% for 98 models). The model-averaged parameter estimates found that the ear, joints, and ventral skin had significantly higher spirochete loads than the bladder (Fig. 2; Table S14 in the ESM). The Finnish bank voles had significantly higher spirochete loads in their tissues than the Swiss bank voles (Fig. 2; Table S14 in the ESM). Finally, the interaction between experiment and organ was significant because the difference in ear tissue spirochete load between Finnish and Swiss bank voles was higher than the difference for the other tissues (Fig. 2; Table S14 in the ESM).

### Strain-specific qPCR to determine infection success of the local strain

For the sample of infected Swiss bank voles, 88.2% (30/34) were infected with the local strain of *B*. *afzelii* (strain NE4049). For the sample of infected Finnish bank voles, 98.0% (49/50) were infected with the local strain of *B*. *afzelii* (strain Fin-Jyv-A3). Thus, 94.0% (79/84) of the bank voles became infected with their local (and intended) strain of *B*. *afzelii* following the infectious challenge.

## Discussion

In this study, we standardized exposure to infected ticks and tested whether a genetic polymorphism at the TLR2 locus in the bank vole explained variation in susceptibility to *B*. *afzelii*, a common and important tick-borne pathogen. Most studies on the role of TLR2 polymorphisms in the susceptibility to *B*. *burgdorferi* sl pathogens have been association studies in humans and wild rodents^4,13,14,26,39,40^. To our knowledge, there are no experimental infection studies showing that a TLR2 polymorphism influences variation in susceptibility to infection with *B*. *burgdorferi* sl. Under the laboratory conditions of our study, ~100% of all bank voles become infected following exposure to *B*. *afzelii*-infected ticks. Thus, our study found no evidence that bank voles with different TLR2 genotypes (C1C1, C2C2, C3C3) differed in their susceptibility to infection with *B*. *afzelii*. One limitation of this study was the small sample size for the C2C2 genotypes (n = 12), which limits our power to detect differences in susceptibility to *B*. *afzelii* infection between the susceptible (C1C1) and resistant (C2C2) genotypes.

The prevalence of *B*. *afzelii* infection in our study (95.5%) was 2.5 times higher than the field study by Tschirren *et al*. (37.5%)^13^ as shown in Fig. 1. This discrepancy is due to differences in the risk of exposure between the two studies. In our study, the risk of exposure was 100.0% for the subset of 88 bank voles that had been successfully challenged (by definition). In the field study by Tschirren *et al*.^13^, the risk of exposure was unknown, but was assumed to be identical for the three TLR2 genotypes. Under this assumption and after minimizing the risk of exposure (subject to the assumption that bank voles cannot clear their infection), the susceptibility of the resistant C2C2 TLR2 genotype remained significantly lower in the study by Tschirren *et al*.^13^ compared to our study (Fig. 1). Why did we not find the expected differences in susceptibility to infection with *B*. *afzelii* between the TLR2 genotypes?

One explanation for the different infection prevalences in the field study by Tschirren *et al*.^13^ is that the three TLR2 genotypes differed in their risk of exposure to *B*. *afzelii*-infected nymphs. Fine-scale spatial studies have shown that the density of infected nymphs (DIN) and the risk of exposure can vary dramatically over very small distances^41^. If by chance, families of bank voles carrying the C2 allele live in areas of the sampling grid that have a low DIN compared to families of bank voles carrying the C1 allele, then observed differences in infection prevalence would be caused by these spatial differences in the risk of exposure and not by differences in susceptibility between the TLR2 genotypes. Differences in exposure can also explain why the prevalence of *B*. *afzelii* infection was 2.5 times higher in our lab study compared to the field study by Tschirren *et al*.^13^.

A second explanation involves grooming, which is an important behaviour that protects vertebrate hosts against ectoparasites such as ticks^42^. Previous studies have shown that the probability of nymph-to-host transmission of *B*. *burgdorferi* sl pathogens increases with the duration of attachment of the nymphal tick to the vertebrate host^43,44^. In nature, the normal grooming behaviour of the vertebrate host is a significant cause of tick mortality that would reduce the risk of pathogen transmission following nymphal attachment^42,45^. In the present study, we prevented the bank voles from grooming off the nymphs by fitting them with a collar and by placing the nymphs in a protective capsule. If C2C2 individuals have a more effective grooming response than C1C1 individuals, they would be less likely to become infected with *B*. *afzelii* following the attachment of an infected nymph. The link between the immune system and grooming behaviour is not so far-fetched because the former has itch-generation mechanisms (e.g. release of histamine by mast cells) that would stimulate the latter^46^. The absence of the grooming defence mechanism can also explain why the prevalence of *B*. *afzelii* infection was 2.5 times higher in our lab study compared to the field study by Tschirren *et al*.^13^.

A third explanation involves acquired immunity, which can influence susceptibility to infection with *B*. *afzelii*. We have recently shown that infected female bank voles can transmit maternal antibodies to their offspring that protect against infection with *B*. *afzelii*^47–49^. Bank voles can also develop acquired immunity against *I*. *ricinus* ticks^50^, and this anti-tick immunity protects against infection with *B*. *burgdorferi* sl^51–53^. Thus variation in maternal antibodies and/or anti-tick immunity in wild bank vole populations will influence variation in susceptibility to infection with *B*. *burgdorferi* sl. In our study, the bank voles did not have any antibodies to either *B*. *afzelii* or to *I*. *ricinus* ticks at the time of the infectious challenge (i.e. they were completey naive). In contrast, field studies can not control for this variation in acquired immunity^13,14,26^. The absence of maternal antibodies or anti-tick immunity can also explain why the prevalence of *B*. *afzelii* infection was 2.5 times higher in our lab study compared to the field study by Tschirren *et al*.^13^

A fourth explanation involves the ability of bank voles to clear *B*. *afzelii*. The consensus is that rodent reservoir hosts cannot clear *B*. *burgdorferi* sl^18–20,23,27,54^. In our study, there was no evidence of clearance at 49 days post-infection, as the four tissues in the 84 infected bank voles were all positive for *B*. *afzelii*. However, we recently found clearance of spirochetes from ear tissues in bank voles that had been experimentally infected with *B*. *afzelii* strain NE4049^48^. Bank voles that had cleared the infection from their ear tissues still had very high spirochete loads in other tissues (bladder, heart, dorsal skin, ventral skin)^48^. This study suggests that ear tissue biopsies might underestimate the infection status of bank voles in the field^48^. In most field studies, the infection status of the bank voles is determined by taking ear tissue biopsies^13,14,26^. Thus, another explanation for the difference in prevalence of *B*. *afzelii* infection between the two studies, is that we determined infection status by testing for spirochetes in multiple internal organs.

A fifth explanation is that our laboratory environment was so stressful that it overwhelmed the variation in susceptibility to infection between the TLR2 genotypes. Physiological stress can suppress the host immune system and increase susceptibility to infection with pathogens^55–58^. Although we cannot rule out the stress-immunosuppression explanation, we believe that it is unlikely for the following reasons. First, there is a long history in Lyme disease research of performing experimental infections with wild animals in the lab, and this work has successfully determined the susceptibility of many vertebrate hosts to various *B*. *burgdorferi* sl pathogens^18,19,59–65^. Second, as mentioned previously, using the same experimental conditions described in the present study, female bank voles infected with *B*. *afzelii* transmitted antibodies to their offspring, which protected the offspring against infected ticks^47–49^. The creation of antibodies in the mothers and their ability to block infection in the offspring requires the coordinated action of both the adaptive and innate immune system. That study provides some evidence that the immune system of our bank voles is functioning as it should under our laboratory conditions^47–49^. Third, although the laboratory environment may be stressful, other stressors found in nature, such as predators, food shortage, competitive interactions, and other parasites, were absent in our lab environment. Fourth, lab mice (*Mus musculus*) are presumably not stressed by our lab conditions, but they are also 100% susceptible to *B*. *afzelii*^29^. The most parsimonious explanation is that competent rodent reservoir hosts are highly susceptible to infection with *B*. *burgdorferi* sl if *Ixodes* nymphs feed to completion.

A sixth explanation for the difference in susceptibility to *B*. *afzelii* infection between our laboratory study and the field study by Tschirren *et al*.^13^ is the presence of other parasites. In natural systems, hosts are often co-infected with multiple parasites including viruses, bacteria, protozoans, helminths, and ectoparasites^66,67^. Field studies have shown that the susceptibility of infection to any particular parasite depends on the presence of other parasites^68^. For example, in a wild population of the field vole (*Microtus agrestis*), infection with tick-borne *Babesia microti* increased and decreased the risk of acquiring infections with tick-borne *Anaplasma phagocytophilum* and flea-borne *Bartonella* spp., respectively^68^. A recent experimental infection study using lab mice found no evidence that infection with the nematode *Heligmosomoides polygyrus* influenced the susceptibility to tick-borne *B*. *afzelii*^69^. In our laboratory system, co-infection with other parasites cannot increase the susceptibility of naive bank voles to tick-borne *B*. *afzelii* because this susceptibility is already very high (95.5%). However, we cannot exclude that co-infection with other parasites in nature decreases the susceptibility to tick-borne *B*. *afzelii*, but to our knowledge, no such parasite has been identified.

A unique aspect of this study was our effort to expose the bank voles to a realistic infectious challenge. Field studies have shown that rodents are rarely infested with more than one *I*. *ricinus* nymph at a time^13,23,24^. In the present study, we collected between 1 and 2 engorged *B*. *afzelii*-infected nymphs from each bank vole indicating that they had been exposed to an ecologically relevant infectious tick bite challenge. We collected more engorged *B*. *afzelii*-infected nymphs per bank vole in the Finnish infection experiment (1.92) than the Swiss infection experiment (1.24). The reason for this difference was improved methodology; the consistent use of collars in the Finnish infection experiment prevented the bank voles from removing the capsules and from grooming off and killing the ticks. The improvement in the infestation methodology explains why the infection success was higher in the Finnish experiment than the Swiss experiment. However, when the infection success was restricted to the subset of 88 bank voles that had been successfully challenged, the infection success was similar between the two experiments. Most important for the purpose of this study was our demonstration that within each infection experiment, the TLR2 genotypes were exposed to the same infectious tick bite challenge.

Estimates of the probability of nymph-to-host transmission of *B*. *burgdorferi* sl pathogens for wild reservoir hosts are rare in the scientific literature^29,31^. Under the conditions of our study, we found that bank voles were highly susceptible to acquiring *B*. *afzelii* infection following an infected tick bite. Of the 88 voles that had been successfully challenged, 95.5% developed a systemic infection (95% CI = 88.1–98.5%). We recently showed for *B*. *afzelii* strain NE4049 that 96.4% of laboratory mice developed a systemic infection following an infected tick bite^29^. Taken together, these studies suggest that tick-to-host transmission of *B*. *afzelii* is ~100% when one or a few infected nymphs feed to completion on a competent rodent host.

The TLR2 receptor plays an important role in controlling the spirochete load in the tissues of laboratory mice (*Mus musculus*). Genetically modified mice that do not have TLR2 have spirochete loads that are 100-fold higher than wild-type mice^9^. We therefore expected resistant C2C2 genotypes to have lower spirochete loads than susceptible C1C1 genotypes. However, our study found no evidence that the TLR2 polymorphism influences variation in the tissue spirochete load of *B*. *afzelii*. The higher tissue spirochete loads in the Finnish versus the Swiss experiment may be due to differences between the two bank vole populations or between the strains of *B*. *afzelii*. Our recent finding that strain Fin-Jyv-A3 establishes a higher tissue spirochete load than strain NE4049 in inbred lab mice^70^ suggests that strain is part of the explanation.

We also found significant differences in spirochete load between organs (Fig. 2). In both infection experiments, the spirochete loads were significantly higher in the ear and the dorsal skin compared to the bladder. These results are similar to a study on *B*. *afzelii* strain NE4049 in lab mice, which found that the spirochete loads were 1.4 to 2.1 times higher in the skin compared to the bladder^29^. Others and we have shown that the probability of host-to-tick transmission of *B*. *afzelii* increases with the spirochete load in the skin^30,71^. *B*. *burgdorferi* sl must establish a persistent infection in the skin to achieve effective host-to-tick transmission^71–74^. *B*. *burgdorferi* sl also invades joint tissues, which causes swelling and arthritis in the ankle joints of lab mice^35,36^. We found no evidence that infection with *B*. *afzelii* caused swelling of the ankle joints in bank voles (see section 7 in the ESM).

We chose to work with *B*. *afzelii* strains NE4049 and Fin-Jyv-A3 because these strains occur in the Swiss and Finnish bank vole populations and because our previous work had shown that they are competent at establishing infection in laboratory mice^29–32^. While the original study by Tschirren *et al*.^13^ did not suggest that the variation in susceptibility to *B*. *afzelii* among TLR2 genotypes would be strain-dependent, we acknowledge that repeating our study with different strains of *B*. *afzelii* might reveal host variation in susceptibility. In each of the two infection experiments, the bank voles were challenged with nymphs that were co-infected with two different strains of *B*. *afzelii*. Co-infections are the norm in nature; 80% of wild *I*. *ricinus* nymphs are infected with multiple strains^75–77^. Likewise, small mammal hosts (including bank voles) are often infected with multiple strains of *B*. *afzelii*^25,78–81^. Thus, co-infected nymphs frequently bite bank voles in nature. In our study, the bank voles were simultaneously challenged with one local strain and one foreign strain. We showed that the local co-adapted strain was present in at least 94.0% (79/84) of the infected bank voles. One potential criticism of our study is that the presence of the foreign strain could have enhanced the ability of the local strain to establish infection. We believe that this explanation is unlikely for two reasons. First, we recently conducted two other studies where Swiss bank voles were experimentally infested with nymphs that were singly infected with either strain NE4049 (n = 22) or strain Fin-Jyv-A3 (n = 21) and found that 95.3% (41/43) of the bank voles became infected^48^. These studies suggest that the susceptibility of bank voles to strains NE4049 and Fin-Jyv-A3 is ~100% and does not depend on whether the nymph is infected with one strain or two strains. Second, we recently found that when lab mice are simultaneously infected with two strains (NE4049 and Fin-Jyv-A3), there can be interference but not facilitation^32^. In summary, we have consistently found that our bank voles and lab mice are ~100% susceptible to infection with *B*. *afzelii* when we successfully challenge them with at least 1 infected nymph.

In conclusion, after controlling for exposure to infected ticks, we did not find that bank voles with different TLR2 genotypes differed in their susceptibility to infection with *B*. *afzelii* under the laboratory conditions of our study. Bank voles were highly susceptible to acquiring *B*. *afzelii* infection when nymphs were allowed to feed to repletion. Future studies should investigate whether different strains of *B*. *afzelii* or different experimental conditions (e.g. semi-natural enclosures that reduce stress) could reveal the expected variation in susceptibility to *B*. *afzelii* infection among TLR2 genotypes. Our study emphasizes the importance of using controlled experimental infections to separate variation in exposure from variation in susceptibility in the study of candidate immune genes for resistance to pathogens.

## Supplementary information

Supplementary Material

Dataset 1

Dataset 2
