# Supplementary material for: Susceptibility to infection with Borrelia afzelii and TLR2 polymorphism in a wild reservoir host
Source: Sci Rep. 2019 Apr 30;9:6711. doi: 10.1038/s41598-019-43160-3 (PMC6491475; doi:10.1038/s41598-019-43160-3)
Supplement: Supplementary file 1 — Supplementary Material [file 41598_2019_43160_MOESM1_ESM.docx]

Title: Susceptibility to infection with *Borrelia afzelii* and TLR2 polymorphism in a wild reservoir host

Authors: Andrea Gomez-Chamorro, Florian Battilotti, Claire Cayol, Tapio Mappes, Esa Koskela, Nathalie Boulanger, Dolores Genné, Anouk Sarr, and Maarten J. Voordouw

Electronic supplementary material (ESM)

**Section 1 – Field site for trapping wild bank voles in Switzerland**

Two trapping sessions were performed at two different sites in Switzerland. The first trapping session was performed in the summer of 2014 at a site above the city of Neuchâtel (47°00'21.9"N 6°56'33.9"E). The second trapping session was performed in the summer of 2015 at a site above the city of Zurich in Zürichbergwald (47°23'41.2"N 8°33'43.0"E). The Neuchatel site was chosen for its high abundance of rodents and *B. afzelii*-infected nymphs ^1^. The Zurich site was chosen because a previous study had shown that the frequency of the C2 resistance allele was high in the local bank vole population ^2^. A total of 110 traps were placed along transects at each field site. The traps were baited with apple and oatmeal and were checked three times per day. All captured bank voles were transported to the animal facility of the University of Neuchâtel.

**Section 1 – Field trapping and lab rearing wild bank voles in Finland**

The bank vole breeding and rearing conditions in laboratory are detailed in ^3^. The lab colony of the University of Jyväskylä is composed of descendants of wild-caught individuals, mainly first and second generation. Wild bank voles are caught within the framework of a long-term population-monitoring program; sampling occurs four times per year (in May, July, August, November) in the Konnevesi area in Central Finland. In each of 20 monitored forests, four Ugglan multiple-capture traps are placed at the corners of a 15-meter square, baited with sunflower seeds for two consecutive days and checked daily. Trapped bank voles are taken to the laboratory facility at the University of Jyväskylä where they measured and kept in standard mouse cages (43 cm*26 cm*15 cm) with wood shavings and under 16 h light:8 h dark photoperiod and temperature of ~19 °C. Animals have *ad libitum* access to food and water ^4^.

Initial matings took place during the spring of 2016 and the founders (F0 voles) were born between the end of January and March 2016. Offspring were separated from their mothers after weaning (21 days) and were kept in sibling groups according to their sex. Each individual was identified with a microchip inserted subcutaneously. After weaning, all individuals were measured, sampled for TLR2 genotyping and housed individually. Similarly, two directional mating sessions, based on TLR2 genotype, which aimed at maximising the number of individuals with C1C1, C2C2 and C3C3 genotypes, took place during the spring of 2016 and created the F1 and F2 generations. Bank voles from the F0, F1 and F2 generations were used in the experimental challenge.

**Section 2 – TLR2 genotyping of the bank voles**

The TLR2 genotype of each individual bank vole was determined as follows. An ear biopsy was taken from each bank vole with a punch type forceps (2 mm in diameter). Total DNA was extracted from the bank vole ear biopsies using the QIAGEN DNeasy® Blood and Tissue Kit and following the manufacturer’s instructions. The DNA was eluted in a final volume of 150 µl of AE buffer and stored at –20°C. The DNA concentration of each sample was measured using a Nanodrop® 2000. The PCR protocol targeted a 1200 base pair fragment of the TLR2 gene and was described previously ^5^. PCR reactions were performed in a total volume of 25 µL including 25 ng of total genomic DNA, 1x Green GoTaq® Reaction Buffer (Promega AG), 0.125 mM dNTPs, 1 mM of each primer (forward and reverse) and 2.5 U of Go TAQ polymerase (Promega AG). The thermocycler conditions were as follows: initial denaturation step at 94°C for 2 min, 37 cycles of denaturation at 94°C for 30 sec, annealing at 59°C for 30 sec, and extension at 72°C for 90 sec, followed by a final extension step at 72°C for 10 min. PCR products were visualized by gel electrophoresis (0.8% agarose gel dyed with Midori Green) and then sent to Microsynth AG for forward and reverse sequencing.

**Section 2 – Assignment of TLR2 sequences to haplotypes and clusters**

The TLR2 sequences were assigned to haplotypes and to the three clusters: C1, C2, and C3. Only high quality sequences were used for haplotype reconstruction. Forward and reverse TLR2 sequences were processed, assembled, and aligned using Geneious version 6.1 (http://www.geneious.com). Consensus sequences were created and polymorphisms were examined visually. Database searches and sequence comparisons were performed with the BLAST tool provided by the National Center for Biotechnology. TLR2 haplotypes were reconstructed using the default settings of the PHASE v2.1 software ^6^. Input files were created using the SeqPHASE web tool ^7^. The alignment of all the individuals was processed in TCS v1.21 to construct the TLR2 haplotype network ^8^.

**Section 2 – Frequency of the TLR2 alleles in the wild bank vole populations**

For the Swiss bank voles, a total of 36 and 59 wild bank voles were captured at the field sites of Neuchâtel and Zurich, respectively. In Neuchatel, the frequencies of the C1 and C3 clusters were 42.2% and 57.8%, respectively, whereas in Zurich, the frequencies of the C1 and C3 clusters were 65.2% and 34.7%, respectively. For the Neuchatel and Zurich samples, 8.6% (3/35) and 18.6% (11/59) of the animals were infected with *B. afzelii* at the time of capture. In Neuchatel, the 3 bank voles infected with *B. afzelii* had the following TLR2 genotypes: 1 C1C1, 1 C1C3, and 1 C3C3. In Zurich, the 11 bank voles infected with *B. afzelii* had the following TLR2 genotypes: 4 C1C1, 6 C1C3, and 1 C3C3. Therefore, there was no association between *B. afzelii* infection and the TLR2 genotype in the Neuchatel and Zurich populations*.*


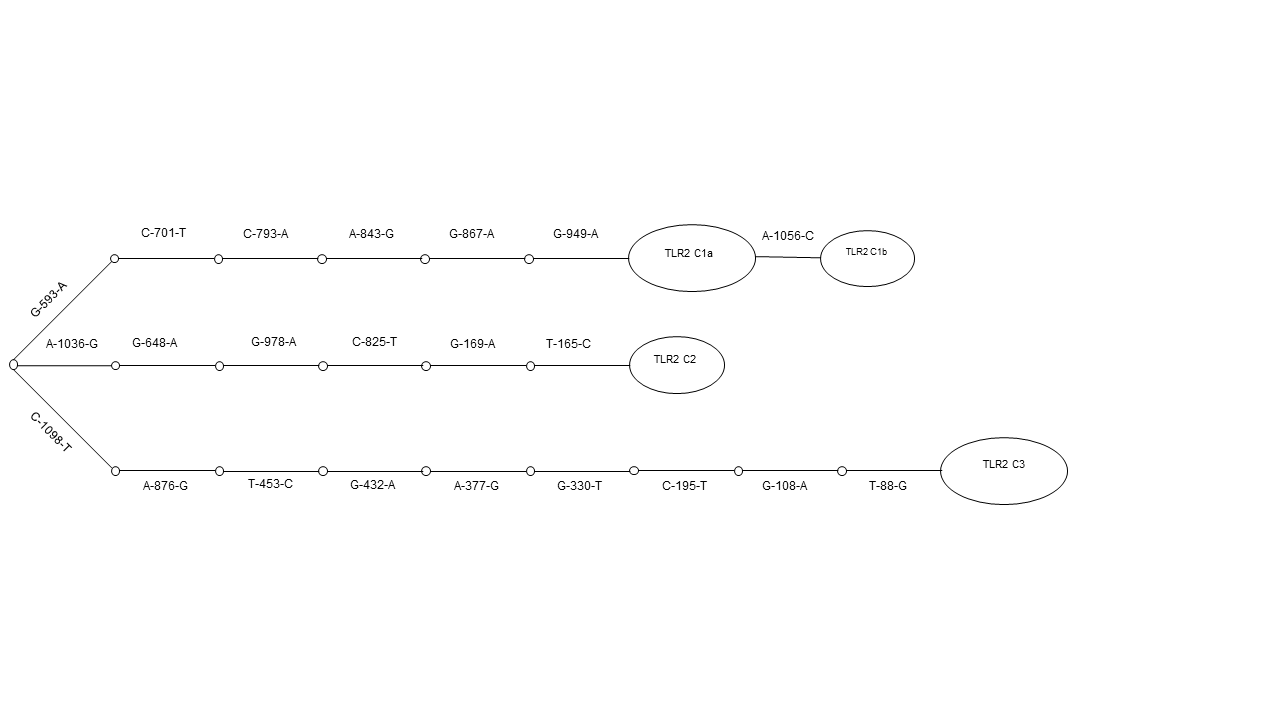


**Figure S1.** TLR2 haplotype network is shown for the 50 Swiss and 50 Finish bank voles that were included in both infection studies. The C1 and C2 clusters, C1 and C3 clusters, and C2 and C3 clusters were separated by a genetic distance of 12, 15, and 15 nucleotides, respectively, which corresponds to a protein distance of 7, 7, and 4 amino acids, respectively. The lines connecting the nodes indicate the number and types of nucleotide changes that separate these four TLR2 haplotypes from each other. For example, TLR2 C1a and TLR2 C1b are separated by a single substitution; the label “A-1056-C” indicates that at site 1056, TLR2 C1a has an adenine whereas TLR2 C1b has a cytosine.

**Section 3 – Isolates of *B. afzelii***

Our original intention was to challenge the Swiss and Finnish bank voles with ticks carrying a single Swiss isolate (NE4049) and a single Finnish isolate (Fin-Jyv-A3) of *B. afzelii*, respectively. We chose these two strains because our previous work had shown that they are highly infectious to laboratory mice ^9-12^. Due to a combination of experimental error and time constraints, we used nymphs that were co-infected with two strains of *B. afzelii*. The Swiss bank voles were challenged with nymphs co-infected with a local Swiss strain (NE4049) and a foreign Austrian strain (E61). The Finnish bank voles were challenged with a local Finnish strain (Fin-Jyv-A3) and a foreign Swiss strain (NE4049).

Isolates NE4049, Fin-Jyv-A3, and E61 were originally obtained from an *I. ricinus* tick in Neuchâtel, Switzerland, a bank vole in Jyväskylä, Finland, and a human patient in Austria, respectively. We have characterized these isolates genetically ^3,10,13^. Fin-Jyv-A3 has *ospC* major group (oMG) A3, multi-locus sequence type (MLST) 676, and strain ID number 1961 in the *Borrelia* MLST database. Isolate NE4049 has oMG A10, MLST 679, and strain ID number 1887 in the *Borrelia* MLST database. Isolate E61 has oMG A3, MLST ST75, and strain ID number 1888 in the *Borrelia* MLST database.

**Section 3 – Creation of nymphs infected with *B. afzelii***

The *B. afzelii*-infected nymphs used to challenge the bank voles were created as follows. Female, *Borrelia*-free *Mus musculus* BALB/c mice were infected with isolates of *B. afzelii* via tick bite. Four weeks after infection, each *B. afzelii*-infected mouse was infested with ~100 *Borrelia*-free, larval ticks from our laboratory colony of *I. ricinus* at the University of Neuchâtel. Blood-engorged larvae were placed in individual tubes and were allowed to moult into *B. afzelii*-infected nymphs. For the Swiss and Finnish infection experiments, the prevalence from a random sample of *B. afzelii*-infected nymphs was 91.3% (42 infected nymphs/46 total nymphs) and 96.8% (91 infected nymphs/94 total nymphs), respectively.

**Section 3 – Strain-specific qPCR to determine infection success of the local isolate**

The Swiss bank voles were challenged with a local Swiss isolate (NE4049) and a foreign Austrian isolate (E61). The Finnish bank voles were challenged with a local Finnish isolate (Fin-Jyv-A3) and a foreign Swiss isolate (NE4049). For the purpose of the experiment, it was important to show that the bank voles had become infected with their local isolates. We had previously developed a qPCR protocol that allows us to differentiate between *B. afzelii ospC* major group (oMG) alleles A10 and A3 ^12,14^. This strain-specific qPCR uses general primers to amplify the *ospC* gene but uses different probes to detect the two oMG alleles ^12,14^. This qPCR can be used to differentiate between isolates NE4049 and E61, which carry oMG alleles A10 and A3, respectively, and between isolates Fin-Jyv-A3 and NE4049, which carry oMG alleles A3 and A10, respectively. We performed this qPCR protocol to determine the infection success of the local isolate.

**Section 3 - *Borrelia afzelii* infection status of the engorged nymphs and the bank voles**

DNA extractions from engorged nymphs and bank vole tissue samples were performed using the 96-well plate QIAGEN DNeasy® Blood and Tissue Kit and following the manufacturer’s instructions. Engorged nymphs were crushed using a sterile steel bead in a Tissue Lyser (Peqlab, Erlangen, Germany). For each bank vole organ, ~20 to 25 mg of tissue was used for DNA extraction and the elution volume was 200 μl. Each of the 6 DNA extraction plates included 12 tissue samples of uninfected lab mice as negative controls (total of 72 negative controls). We measured the DNA concentration of each DNA extraction using a Nanodrop machine. The DNA concentration is an estimate of the amount of host DNA and we used it to standardize our estimates of spirochete *B. afzelii* load per mg of host DNA (see below).

The *B. afzelii* infection status and spirochete load of the engorged nymphs and the tissue samples of the bank vole organs were estimated using a qPCR assay that amplified a 132 bp fragment of the *flagellin* gene ^15^. The qPCR reaction was performed following the protocol of Jacquet et al. ^10^; 3 μl of DNA template were used per sample. Each qPCR plate contained 81 samples from individual bank voles (and the tissue samples from the lab mice that served as negative DNA extraction controls), and triplicates of standards (10, 100, 1000, 10000 *flagellin* gene copies) and 3 negative controls for the qPCR (template was 3 μl of distilled water). Each bank vole tissue sample was replicated in three independent qPCRs. The reactions were run in the LightCycler® 96 (Roche Applied Science, Switzerland). The number of spirochetes present in each engorged nymph and bank vole tissue sample was calculated using the standard curves and the LightCycler® 96 software (Roche Applied Science, Switzerland). All of the 72 negative controls for the DNA extraction (tissue samples from lab mice) tested negative for *B. afzelii*.

**Table S01.** Infection status is shown for the 50 Swiss bank voles in the Swiss infection experiment. Bank voles were considered as having been successfully challenged if we collected at least 1 *B. afzelii*-infected engorged nymph from the capsule (Engorged nymphs = Positive) or if the individual developed a systemic infection (Vole infection = Positive). Individuals for which there was no proof that they had been exposed to at least one *B. afzelii*-infected engorged nymph (Engorged nymphs = Negative; Vole infection = Negative) were excluded from the experiment (12 animals in the top row in Table S1).

| Engorged  nymphs | Vole  infection | Challenged | C1C1 | C1C3 | C3C3 | Total |
| --- | --- | --- | --- | --- | --- | --- |
| Negative | Negative | No | 3 | 6 | 3 | 12 |
| Negative | Positive | Yes | 2 | 0 | 2 | 4 |
| Positive | Negative | Yes | 3 | 0 | 1 | 4 |
| Positive | Positive | Yes | 9 | 9 | 12 | 30 |
|  |  |  | 17 | 15 | 18 | 50 |

**Table S02.** Infection status is shown for the 50 Finnish bank voles in the Finnish infection experiment. Bank voles were considered as having been successfully challenged if we collected at least 1 *B. afzelii*-infected engorged nymph from the capsule (Engorged nymphs = Positive) or if the individual developed a systemic infection (Vole infection = Positive). Individuals for which there was no proof that they had been exposed to at least one *B. afzelii*-infected engorged nymph (Engorged nymphs = Negative; Vole infection = Negative) were excluded from the experiment (0 animals in the top row in Table S2).

| Engorged  nymphs | Vole  infection | Challenged | C1C1 | C1C3 | C3C3 | C1C2 | C2C2 | C2C3 | Total |
| --- | --- | --- | --- | --- | --- | --- | --- | --- | --- |
| Negative | Negative | No | 0 | 0 | 0 | 0 | 0 | 0 | 0 |
| Negative | Positive | Yes | 0 | 0 | 1 | 0 | 0 | 0 | 1 |
| Positive | Negative | Yes | 0 | 0 | 0 | 0 | 0 | 0 | 0 |
| Positive | Positive | Yes | 11 | 8 | 9 | 8 | 12 | 1 | 49 |
|  |  |  | 11 | 8 | 10 | 8 | 12 | 1 | 50 |

**Section 4 – Correspondence between field and lab estimates of susceptibility to infection:**

The probability that a bank vole is infected with *B. afzelii* in nature (P_I_) is the probability of exposure to an infectious challenge (P_E_) times the probability of acquiring an infection following an infectious challenge, hereafter referred to as the susceptibility (P_S_) ^16^. In the field, P_I_ = P_E_*P_S_. In our study, we controlled the probability of exposure (i.e. P_E_ was set to 1.000) and we were therefore able to estimate the susceptibility to infection (P_S_). After combining the data from our two infection experiments, the susceptibility to infection for TLR2 genotypes C1C1, C1C2, and C2C2 was 88.0% (22/25), 100.0% (8/8), and 100.0% (12/12), respectively.

In the study by Tschirren et al. ^17^, the prevalence of *B. afzelii* infection of adult bank voles in the field for TLR2 genotypes C1C1, C1C2, and C2C2 was 48.6% (72/148), 30.6% (48/157), and 17.9% (5/28), respectively. An important assumption in the study by Tschirren et al. ^17^ was that the probability of exposure (P_E_) was the same for all three genotypes. If we assume that the probability of exposure is 100% (i.e. as in our lab experiment), then the susceptibility of infection is equal to the observed prevalence of infection and is 48.6%, 30.6%, and 17.9% for the TLR2 genotypes C1C1, C1C2, and, respectively. However, this assumption is not realistic. We can set a lower limit for the probability of exposure, by assuming that the C1C1 genotype is 100% susceptible. In this case, the probability of exposure is 48.6% and the susceptibility of infection is 100.0%, 62.8%, and 36.7% for TLR2 genotypes C1C1, C1C2, and C2C2 (**Table S03**).

We now ask, what is the probability that the susceptibilities in the study by Tschirren et al. ^17^ could have generated the observed susceptibilities in our laboratory study? We can use the binomial probability functions in R to calculate the probabilities that the susceptibilities in the field could have generated the observed susceptibilities in the lab as follows:

C1C1: 1-pbinom(21, 25, 1.000000) = 1.000

C1C2: dbinom(8, 8, 0.6284501) = 0.02433136

C2C2: dbinom(12, 12, 0.3670634) = 0.000005982642

**Table S03**. The probability that a bank vole is infected with *B. afzelii* (P_I_) is the product of the probability of exposure to an infectious challenge (P_E_) and the probability of acquiring an infection following an infectious challenge (Probability of susceptibility = P_S_). Two opposite scenarios are shown where the probability of exposure is either maximal (100.0%) or minimal (48.6%). Under the minimal probability of exposure, the C1C1 genotype is maximally susceptible (100.0%).

| TLR2 | P_E_ | P_S_ | P_I_ |
| --- | --- | --- | --- |
| C1C1 | 100.0% | 48.6% | 48.6% |
| C1C2 | 100.0% | 30.6% | 30.6% |
| C2C2 | 100.0% | 17.9% | 17.9% |
| C1C1 | 48.6%, | 100.0% | 48.6% |
| C1C2 | 48.6%, | 62.8% | 30.6% |
| C2C2 | 48.6%, | 36.7% | 17.9% |

Under the assumption of independence, the probability that the field susceptibilities estimates could generate the laboratory data is the product of the above probabilities: 2*1.000*0.02433136*0.000005982642 = 0.0000002911316. In the preceding equation, we doubled this probability because we had no ‘a priori’ expectation of whether the lab susceptibilities would be more or less extreme than the field susceptibilities (i.e. were are doing a two-tailed probability test). This exercise shows that it is very improbable (less than 1 in a million) for the field susceptibility estimates to generate our observed experimental data. For this calculation, we made the field and lab susceptibility estimates as close as possible to each other by assuming that the probability of exposure was as low as possible (48.6%).

**Section 5 – Repeatability of the *B. afzelii* spirochete loads in the tissues of the bank voles**

The spirochete load in the tissues of the bank voles was estimated using a qPCR assay that targeted the *flagellin* gene of *B. afzelii*. For each tissue sample, the *flagellin* gene copy number is an estimate of the spirochete load. The repeatability is a commonly used metric to assess the reliability of scoring a particular phenotype. We estimated the repeatability for two phenotypes associated with the spirochete load in the tissues of the bank voles: the quantification cycle (Cq) and the spirochete load (SL; first divided by the DNA concentration of the DNA extraction of the tissue sample, then log10-transformed). We used a generalized linear mixed effects model to estimate the ‘among’ and ‘within’ variance components of the Cq and of the SL. These variance components were used to calculate the repeatability of the Cq and the SL (**Table S04**).

**Table S04.** Repeatability of the quantification cycle (Cq) and the repeatability of the spirochete load (SL) are shown for each organ.

| Organ | N | Repeatability  of Cq | Repeatability  of SL |
| --- | --- | --- | --- |
| Heart | 54 | 82% | 59% |
| Joint | 96 | 96% | 71% |
| Skin | 93 | 89% | 53% |
| Ear | 99 | 95% | 59% |
| Total | 342 | 97% | 75% |

**Section 6 – Re-analysis of the *B. afzelii* infection prevalence data by Tschirren et al. ^17^**

The bank voles in the field study by Tschirren et al. ^17^ were captured during the months of May, June, August, September, and October. Tick questing activity is highly seasonal and it is well established that the risk of exposure to infected ticks varies dramatically over the course of the transmission season ^18^. The prevalence of infection in the bank voles in each month of the study is an index of the risk of infection. In the original statistical analysis in Tschirren et al. ^17^, infection prevalence was modelled as a generalized linear mixed effects model and month was modelled as a random effect. While this approach controls for the variance in infection risk among months, it does not examine how infection prevalence (and risk of infection) changes over time. We therefore re-analysed the data from the study by Tschirren et al. ^17^ to determine how infection prevalence (and risk of infection) varied over the months of the study. The infection prevalence was higher in the months of June, August, and September compared to the months of May and October (**Figure S2**) and these differences were statistically significant (Δ df = 4, Δ χ^2^ = 19.912, p < 0.001).

We used a generalized linear model with binomial errors to model infection prevalence as a function of TLR2 genotype, sex, and their interaction separately for each month. TLR2 genotype was modelled as the number of C2 alleles. We expect the slope of the relationship between infection prevalence and the number of C2 alleles to be negative. The sex:TLR2 genotype interaction was not significant for any of the months and we therefore re-ran the models without this interaction term. The parameter estimates for TLR2 genotype and sex are shown for each month in **Table S05**. The expected negative effect of the number of C2 alleles was statistically significant for three of the five months (June, August, and September). This analysis shows that the differences in infection prevalence between the three TLR2 genotypes are not biased by differences in the risk of infection between months.

**Figure S2.** The prevalence of bank voles infected with *B. afzelii* is shown for each month for the field study by Tschirren et al. ^17^.

**Table S05.** Analysis of the prevalence of *B. afzelii* infection in the bank voles of the field study by Tschirren et al. ^17^. For each month of the field study, infection prevalence was modelled as a function of TLR2 genotype and sex using a GLM with binomial errors. TLR2 genotype was modelled as the number of C2 alleles and sex is modelled as a contrast between males and females. Shown are the parameters, the parameter estimates, the standard errors, the z-values and associated p-values.

| Month | Parameter | Estimate | Std. Error | z-value | p |
| --- | --- | --- | --- | --- | --- |
| May | Intercept | -3.951 | 1.454 | -2.717 | 0.007 |
| May | C2.alleles | 1.644 | 1.125 | 1.462 | 0.144 |
| May | sexM | 1.409 | 1.264 | 1.115 | 0.265 |
| June | Intercept | 0.317 | 0.490 | 0.647 | 0.518 |
| **June** | **C2.alleles** | **-1.486** | **0.480** | **-3.099** | **0.002** |
| June | sexM | 1.020 | 0.532 | 1.919 | 0.055 |
| August | Intercept | 0.120 | 0.336 | 0.358 | 0.720 |
| **August** | **C2.alleles** | **-0.895** | **0.351** | **-2.549** | **0.011** |
| August | sexM | 0.755 | 0.452 | 1.669 | 0.095 |
| September | Intercept | 0.133 | 0.424 | 0.314 | 0.754 |
| **September** | **C2.alleles** | **-1.050** | **0.456** | **-2.304** | **0.021** |
| September | sexM | -0.089 | 0.531 | -0.167 | 0.867 |
| October | Intercept | -0.900 | 0.434 | -2.077 | 0.038 |
| October | TLR2 | -0.122 | 0.450 | -0.271 | 0.786 |
| October | sexM | -0.314 | 0.584 | -0.537 | 0.591 |

**Section 7 – Materials and methods and statistical analysis of the morphometric data**

Infection with *B. burgdorferi* sensu stricto in laboratory mice is known to cause inflammation and/or swelling in organs, such as the heart and ankle joints (Wooten et al., 2002). To test whether *B. afzelii* induces inflammation in a natural reservoir host like the bank vole, we measured a number of morphological variables in the Swiss bank voles. Ankle diameter (mm) was measured for each bank vole prior to sacrifice. The heart weight (mg) and bladder weight (mg) were measured after the bank voles had been sacrificed and frozen. LME models with normal errors were used to test whether infection with *B. afzelii* and TLR2 genotype influenced these morphological variables. The TLR2 genotype (three levels: C1C1, C1C3, C3C3), sex (two levels: males and females), infection status (two levels: infected and uninfected), and the covariate body mass were modelled as fixed factors, whereas the trial number was modelled as a random factor.

**Section 7 – Results of the morphometric data**

For the sample of Swiss bank voles, LME models with normal errors were used to test whether infection with *B. afzelii* and TLR2 genotype influenced the following three phenotypes: heart weight, bladder weight, and ankle diameter. There was no effect of *B. afzelii* infection and no effect of TLR2 genotype on heart weight, bladder weight, or ankle diameter (**Table S06**).

**Table S06.** Morphological measurements of 5 different phenotypes are compared between the uninfected and the *Borrelia*-infected bank voles (mean ± standard deviation) in the Swiss population.

| Organ | Uninfected | Infected |
| --- | --- | --- |
| Ankle Left (mm) | 2.38 ± 0.15 | 2.35 ± 0.17 |
| Ankle Right (mm) | 2.31 ± 0.13 | 2.30 ± 0.12 |
| Heart (mg) | 132.98 ± 22.92 | 148.68 ± 34.89 |
| Bladder (mg) | 27.42 ± 8.94 | 33.21 ± 15.24 |
| Weight (g) | 23.92 ± 4.33 | 25.04 ± 5.44 |

**Table S07.** Model selection table is shown for the generalized linear models (with binomial errors) of the number of engorged nymphs per bank vole (nymphs.engorged). The analysis was done on the entire data set of 100 bank voles. Of the 29 models, the top 3 models have 99.999% of the support. The fixed factors are experiment (E), sex (S), and TLR2 genotype. TLR2 genotype was modelled in 8 different ways (geno1, geno2, geno3, geno4, geno5, geno6, geno7, and geno8). For each model, the model structure, degrees of freedom (df), log likelihood, corrected AIC value (AICc), difference in AICc from the top model (Delta), and the support (Weight) are shown.

| Model | Model Structure | df | logLik | AICc | delta | weight |
| --- | --- | --- | --- | --- | --- | --- |
| model003 | nymphs.engorged~E | 2 | -109.353 | 222.831 | 0.000 | 67.575 |
| model002 | nymphs.engorged~E+S | 3 | -109.316 | 224.881 | 2.051 | 24.235 |
| model001 | nymphs.engorged~E+S+E:S | 4 | -109.315 | 227.051 | 4.221 | 8.189 |
| model010 | nymphs.engorged~geno2 | 3 | -121.250 | 248.751 | 25.920 | 0.000 |
| model022 | nymphs.engorged~geno5 | 2 | -122.638 | 249.399 | 26.569 | 0.000 |
| model019 | nymphs.engorged~C1+C2+C3 | 3 | -122.122 | 250.494 | 27.663 | 0.000 |
| model009 | nymphs.engorged~geno2+S | 4 | -121.250 | 250.922 | 28.091 | 0.000 |
| model021 | nymphs.engorged~geno5+S | 3 | -122.636 | 251.522 | 28.691 | 0.000 |
| model007 | nymphs.engorged~geno1 | 6 | -119.814 | 252.530 | 29.700 | 0.000 |
| model018 | nymphs.engorged~C1+C2+C3+S | 4 | -122.122 | 252.665 | 29.834 | 0.000 |
| model020 | nymphs.engorged~geno5+S+geno5:S | 4 | -122.602 | 253.625 | 30.794 | 0.000 |
| model016 | nymphs.engorged~geno4 | 3 | -124.254 | 254.758 | 31.928 | 0.000 |
| model006 | nymphs.engorged~geno1+S | 7 | -119.811 | 254.839 | 32.009 | 0.000 |
| model028 | nymphs.engorged~geno7 | 2 | -125.477 | 255.077 | 32.247 | 0.000 |
| model008 | nymphs.engorged~geno2+S+geno2:S | 6 | -121.171 | 255.246 | 32.415 | 0.000 |
| model017 | nymphs.engorged~C1+C2+C3+S+C1:S+C2:S+C3:S | 6 | -121.690 | 256.283 | 33.452 | 0.000 |
| model015 | nymphs.engorged~geno4+S | 4 | -124.165 | 256.751 | 33.920 | 0.000 |
| model027 | nymphs.engorged~geno7+S | 3 | -125.422 | 257.094 | 34.263 | 0.000 |
| model026 | nymphs.engorged~geno7+S+geno7:S | 4 | -124.629 | 257.680 | 34.850 | 0.000 |
| model029 | nymphs.engorged~1 | 1 | -128.286 | 258.613 | 35.782 | 0.000 |
| model014 | nymphs.engorged~geno4+S+geno4:S | 6 | -123.368 | 259.639 | 36.809 | 0.000 |
| model004 | nymphs.engorged~S | 2 | -128.226 | 260.576 | 37.746 | 0.000 |
| model025 | nymphs.engorged~geno6 | 2 | -128.273 | 260.669 | 37.838 | 0.000 |
| model013 | nymphs.engorged~geno3 | 3 | -128.015 | 262.281 | 39.450 | 0.000 |
| model024 | nymphs.engorged~geno6+S | 3 | -128.218 | 262.686 | 39.856 | 0.000 |
| model005 | nymphs.engorged~geno1+S+geno1:S | 11 | -119.121 | 263.241 | 40.411 | 0.000 |
| model012 | nymphs.engorged~geno3+S | 4 | -127.956 | 264.333 | 41.502 | 0.000 |
| model023 | nymphs.engorged~geno6+S+geno6:S | 4 | -128.130 | 264.682 | 41.851 | 0.000 |
| model011 | nymphs.engorged~geno3+S+geno3:S | 6 | -127.459 | 267.821 | 44.991 | 0.000 |

**Table S08.** Model-averaged parameter estimates are shown for the generalized linear models (with binomial errors) of the number of engorged nymphs per bank vole. The model selection table contains 29 models. The fixed factors are experiment (expt), sex (sex), and TLR2 genotype. TLR2 genotype was modeled in 8 different ways (geno1, geno2, geno3, geno4, geno5, geno6, geno7, and geno8). For each of the 38 parameter estimates, mean1, mean2, the lower limit (LL) and upper limit (UL) of the 95% confidence interval (CI) for mean2, and the statistical significance (p < 0.05) are shown. Mean1 is averaged over all the models that contain that parameter in the model selection table. Mean2 is averaged over a subset of models that contain that parameter.

| Parameter | Mean1 | Mean2 | 95% LL | 95% UL | Signif |
| --- | --- | --- | --- | --- | --- |
| Intercept | 0.252 | 0.252 | -0.109 | 0.613 | NS |
| **exptFin** | **1.693** | **1.693** | **1.077** | **2.309** | **P<0.05** |
| sexM | -0.024 | -0.074 | -0.649 | 0.501 | NS |
| exptFin:sexM | -0.002 | -0.019 | -1.203 | 1.165 | NS |
| geno2C2Cx | 0.000 | 0.704 | -1.070 | 2.477 | NS |
| **geno2CxCx** | **0.000** | **-1.106** | **-2.126** | **-0.086** | **P<0.05** |
| **geno5** | **0.000** | **-0.741** | **-1.281** | **-0.202** | **P<0.05** |
| C1 | 0.000 | 0.161 | -0.174 | 0.496 | NS |
| **C2** | **0.000** | **0.808** | **0.272** | **1.344** | **P<0.05** |
| C3 | 0.000 | NA | NA | NA | NA |
| geno1C1C2 | 0.000 | 1.423 | -0.125 | 2.971 | NS |
| geno1C1C3 | 0.000 | -0.533 | -1.225 | 0.159 | NS |
| geno1C2C2 | 0.000 | 0.848 | -0.230 | 1.926 | NS |
| geno1C2C3 | 0.000 | 15.180 | -2229.585 | 2259.946 | NS |
| geno1C3C3 | 0.000 | -0.282 | -0.952 | 0.389 | NS |
| geno5:sexM | 0.000 | -0.137 | -1.177 | 0.902 | NS |
| geno4C3Cz | 0.000 | -0.187 | -0.877 | 0.503 | NS |
| geno4CzCz | 0.000 | 0.621 | -0.030 | 1.272 | NS |
| geno7 | 0.000 | 0.323 | -0.025 | 0.670 | NS |
| geno2C2Cx:sexM | 0.000 | -0.095 | -3.616 | 3.425 | NS |
| geno2CxCx:sexM | 0.000 | -0.373 | -2.415 | 1.668 | NS |
| C1:sexM | 0.000 | 0.294 | -0.363 | 0.951 | NS |
| C2:sexM | 0.000 | 0.248 | -0.815 | 1.311 | NS |
| C3:sexM | 0.000 | NA | NA | NA | NA |
| geno7:sexM | 0.000 | 0.377 | -0.219 | 0.974 | NS |
| geno4C3Cz:sexM | 0.000 | 0.125 | -1.213 | 1.463 | NS |
| geno4CzCz:sexM | 0.000 | 0.725 | -0.513 | 1.964 | NS |
| geno6 | 0.000 | 0.029 | -0.295 | 0.354 | NS |
| geno3C1Cy | 0.000 | -0.173 | -0.854 | 0.509 | NS |
| geno3CyCy | 0.000 | 0.031 | -0.616 | 0.678 | NS |
| geno1C1C2:sexM | 0.000 | -0.464 | -3.568 | 2.639 | NS |
| geno1C1C3:sexM | 0.000 | -0.687 | -2.097 | 0.722 | NS |
| geno1C2C2:sexM | 0.000 | -0.128 | -2.339 | 2.084 | NS |
| geno1C2C3:sexM | 0.000 | NA | NA | NA | NA |
| geno1C3C3:sexM | 0.000 | -0.679 | -2.045 | 0.687 | NS |
| geno6:sexM | 0.000 | -0.132 | -0.757 | 0.493 | NS |
| geno3C1Cy:sexM | 0.000 | -0.672 | -2.018 | 0.674 | NS |
| geno3CyCy:sexM | 0.000 | -0.378 | -1.673 | 0.916 | NS |

**Table S09.** Model selection table is shown for the generalized linear models with binomial errors of bank vole infection status (infection). The analysis was done on the entire data set of 100 bank voles. Of the 117 models, the top 41 models have 95.0% of the support. The fixed factors are number of engorged *B. afzelii*-infected nymphs, experiment (E), sex (S), and TLR2 genotype. TLR2 genotype is modelled in 7 different ways (geno1, geno2, geno3, geno4, geno5, geno6, and geno7). For each model, the model structure, degrees of freedom (df), log likelihood, corrected AIC value (AICc), difference in AICc from the top model (Delta), and the support (Weight) are shown.

| Model ID | Model Structure | df | logLik | AICc | delta | weight |
| --- | --- | --- | --- | --- | --- | --- |
| model014 | infection~E+N | 3 | -21.400 | 49.051 | 0.000 | 17.929 |
| model009 | infection~E+S+N | 4 | -20.592 | 49.605 | 0.554 | 13.591 |
| model011 | infection~E+N+E:N | 4 | -21.400 | 51.222 | 2.171 | 6.055 |
| model307 | infection~geno3+S+N+geno3:N | 7 | -18.038 | 51.292 | 2.242 | 5.845 |
| model008 | infection~E+S+N+S:N | 5 | -20.549 | 51.736 | 2.685 | 4.683 |
| model007 | infection~E+S+N+E:N | 5 | -20.592 | 51.822 | 2.771 | 4.485 |
| model006 | infection~E+S+N+E:S | 5 | -20.592 | 51.822 | 2.771 | 4.485 |
| model302 | infection~geno3+S+N+geno3:S+geno3:N+S:N | 10 | -14.771 | 52.014 | 2.964 | 4.074 |
| model311 | infection~geno3+N+geno3:N | 6 | -19.744 | 52.391 | 3.340 | 3.375 |
| model601 | infection~geno6+S+N+geno6:S+geno6:N+S:N+geno6:S:N | 8 | -17.475 | 52.532 | 3.481 | 3.145 |
| model303 | infection~geno3+S+N+geno3:S+geno3:N | 9 | -16.361 | 52.721 | 3.670 | 2.861 |
| model402 | infection~geno4+S+N+geno4:S+geno4:N+S:N | 10 | -15.469 | 53.410 | 4.359 | 2.028 |
| model305 | infection~geno3+S+N+geno3:N+S:N | 8 | -17.934 | 53.450 | 4.400 | 1.987 |
| model407 | infection~geno4+S+N+geno4:N | 7 | -19.328 | 53.873 | 4.823 | 1.608 |
| model701 | infection~geno7+S+N+geno7:S+geno7:N+S:N+geno7:S:N | 8 | -18.148 | 53.878 | 4.827 | 1.605 |
| model005 | infection~E+S+N+E:N+S:N | 6 | -20.549 | 54.001 | 4.950 | 1.509 |
| model004 | infection~E+S+N+E:S+S:N | 6 | -20.549 | 54.001 | 4.950 | 1.509 |
| model003 | infection~E+S+N+E:S+E:N | 6 | -20.592 | 54.087 | 5.036 | 1.445 |
| model403 | infection~geno4+S+N+geno4:S+geno4:N | 9 | -17.311 | 54.621 | 5.571 | 1.106 |
| model015 | infection~S+N | 3 | -24.246 | 54.742 | 5.692 | 1.042 |
| model607 | infection~geno6+S+N+geno6:N | 5 | -22.248 | 55.134 | 6.084 | 0.856 |
| model018 | infection~N | 2 | -25.668 | 55.459 | 6.408 | 0.728 |
| model509 | infection~geno5+S+N | 4 | -23.532 | 55.484 | 6.434 | 0.719 |
| model514 | infection~geno5+N | 3 | -24.645 | 55.540 | 6.490 | 0.699 |
| model606 | infection~geno6+S+N+geno6:S | 5 | -22.455 | 55.549 | 6.498 | 0.696 |
| model411 | infection~geno4+N+geno4:N | 6 | -21.337 | 55.577 | 6.527 | 0.686 |
| model706 | infection~geno7+S+N+geno7:S | 5 | -22.623 | 55.885 | 6.834 | 0.588 |
| model614 | infection~geno6+N | 3 | -24.830 | 55.910 | 6.860 | 0.581 |
| model609 | infection~geno6+S+N | 4 | -23.766 | 55.953 | 6.902 | 0.569 |
| model405 | infection~geno4+S+N+geno4:N+S:N | 8 | -19.222 | 56.026 | 6.975 | 0.548 |
| model002 | infection~E+S+N+E:S+E:N+S:N | 7 | -20.549 | 56.315 | 7.264 | 0.474 |
| model611 | infection~geno6+N+geno6:N | 4 | -24.008 | 56.438 | 7.387 | 0.446 |
| model603 | infection~geno6+S+N+geno6:S+geno6:N | 6 | -21.776 | 56.456 | 7.405 | 0.442 |
| model709 | infection~geno7+S+N | 4 | -24.068 | 56.557 | 7.507 | 0.420 |
| model012 | infection~S+N+S:N | 4 | -24.207 | 56.835 | 7.784 | 0.366 |
| model707 | infection~geno7+S+N+geno7:N | 5 | -23.143 | 56.924 | 7.873 | 0.350 |
| model406 | infection~geno4+S+N+geno4:S | 7 | -20.896 | 57.010 | 7.959 | 0.335 |
| model714 | infection~geno7+N | 3 | -25.380 | 57.010 | 7.959 | 0.335 |
| model301 | infection~geno3+S+N+geno3:S+geno3:N+S:N+geno3:S:N | 12 | -14.771 | 57.129 | 8.078 | 0.316 |
| model605 | infection~geno6+S+N+geno6:N+S:N | 6 | -22.246 | 57.396 | 8.345 | 0.276 |
| model306 | infection~geno3+S+N+geno3:S | 7 | -21.199 | 57.615 | 8.564 | 0.248 |
| model409 | infection~geno4+S+N | 5 | -23.515 | 57.668 | 8.618 | 0.241 |
| model508 | infection~geno5+S+N+S:N | 5 | -23.521 | 57.679 | 8.629 | 0.240 |
| model506 | infection~geno5+S+N+geno5:S | 5 | -23.532 | 57.702 | 8.651 | 0.237 |
| model209 | infection~geno2+S+N | 5 | -23.532 | 57.702 | 8.651 | 0.237 |
| model507 | infection~geno5+S+N+geno5:N | 5 | -23.532 | 57.702 | 8.651 | 0.237 |
| model214 | infection~geno2+N | 4 | -24.645 | 57.711 | 8.661 | 0.236 |
| model511 | infection~geno5+N+geno5:N | 4 | -24.645 | 57.711 | 8.661 | 0.236 |
| model703 | infection~geno7+S+N+geno7:S+geno7:N | 6 | -22.424 | 57.752 | 8.701 | 0.231 |
| model604 | infection~geno6+S+N+geno6:S+S:N | 6 | -22.429 | 57.761 | 8.710 | 0.230 |
| model309 | infection~geno3+S+N | 5 | -23.600 | 57.839 | 8.788 | 0.221 |
| model314 | infection~geno3+N | 4 | -24.731 | 57.884 | 8.833 | 0.217 |
| model608 | infection~geno6+S+N+S:N | 5 | -23.623 | 57.884 | 8.833 | 0.216 |
| model704 | infection~geno7+S+N+geno7:S+S:N | 6 | -22.622 | 58.148 | 9.097 | 0.190 |
| model414 | infection~geno4+N | 4 | -24.906 | 58.233 | 9.182 | 0.182 |
| model401 | infection~geno4+S+N+geno4:S+geno4:N+S:N+geno4:S:N | 12 | -15.469 | 58.524 | 9.473 | 0.157 |
| model708 | infection~geno7+S+N+S:N | 5 | -23.952 | 58.542 | 9.491 | 0.156 |
| model711 | infection~geno7+N+geno7:N | 4 | -25.116 | 58.653 | 9.602 | 0.147 |
| model001 | infection~E+S+N+E:S+E:N+S:N+E:S:N | 8 | -20.549 | 58.680 | 9.629 | 0.145 |
| model602 | infection~geno6+S+N+geno6:S+geno6:N+S:N | 7 | -21.761 | 58.739 | 9.688 | 0.141 |
| model705 | infection~geno7+S+N+geno7:N+S:N | 6 | -23.138 | 59.179 | 10.128 | 0.113 |
| model404 | infection~geno4+S+N+geno4:S+S:N | 8 | -20.894 | 59.370 | 10.320 | 0.103 |
| model408 | infection~geno4+S+N+S:N | 6 | -23.377 | 59.658 | 10.607 | 0.089 |
| model308 | infection~geno3+S+N+S:N | 6 | -23.428 | 59.760 | 10.709 | 0.085 |
| model304 | infection~geno3+S+N+geno3:S+S:N | 8 | -21.107 | 59.797 | 10.746 | 0.083 |
| model505 | infection~geno5+S+N+geno5:N+S:N | 6 | -23.521 | 59.944 | 10.893 | 0.077 |
| model504 | infection~geno5+S+N+geno5:S+S:N | 6 | -23.521 | 59.944 | 10.893 | 0.077 |
| model208 | infection~geno2+S+N+S:N | 6 | -23.521 | 59.944 | 10.893 | 0.077 |
| model503 | infection~geno5+S+N+geno5:S+geno5:N | 6 | -23.532 | 59.967 | 10.916 | 0.076 |
| model702 | infection~geno7+S+N+geno7:S+geno7:N+S:N | 7 | -22.391 | 60.000 | 10.949 | 0.075 |
| model211 | infection~geno2+N+geno2:N | 6 | -24.645 | 62.194 | 13.143 | 0.025 |
| model502 | infection~geno5+S+N+geno5:S+geno5:N+S:N | 7 | -23.521 | 62.258 | 13.208 | 0.024 |
| model207 | infection~geno2+S+N+geno2:N | 7 | -23.532 | 62.281 | 13.230 | 0.024 |
| model206 | infection~geno2+S+N+geno2:S | 7 | -23.532 | 62.281 | 13.230 | 0.024 |
| model114 | infection~geno1+N | 7 | -23.718 | 62.652 | 13.602 | 0.020 |
| model109 | infection~geno1+S+N | 8 | -22.733 | 63.048 | 13.997 | 0.016 |
| model107 | infection~geno1+S+N+geno1:N | 12 | -17.987 | 63.561 | 14.510 | 0.013 |
| model111 | infection~geno1+N+geno1:N | 11 | -19.552 | 64.103 | 15.052 | 0.010 |
| model205 | infection~geno2+S+N+geno2:N+S:N | 8 | -23.521 | 64.623 | 15.573 | 0.007 |
| model204 | infection~geno2+S+N+geno2:S+S:N | 8 | -23.521 | 64.623 | 15.573 | 0.007 |
| model501 | infection~geno5+S+N+geno5:S+geno5:N+S:N+geno5:S:N | 8 | -23.521 | 64.623 | 15.573 | 0.007 |
| model108 | infection~geno1+S+N+S:N | 9 | -22.619 | 65.237 | 16.187 | 0.005 |
| model105 | infection~geno1+S+N+geno1:N+S:N | 13 | -17.883 | 65.999 | 16.948 | 0.004 |
| model016 | infection~E | 2 | -31.343 | 66.811 | 17.760 | 0.002 |
| model203 | infection~geno2+S+N+geno2:S+geno2:N | 9 | -23.532 | 67.063 | 18.012 | 0.002 |
| model106 | infection~geno1+S+N+geno1:S | 12 | -20.253 | 68.092 | 19.041 | 0.001 |
| model013 | infection~E+S | 3 | -30.998 | 68.247 | 19.196 | 0.001 |
| model202 | infection~geno2+S+N+geno2:S+geno2:N+S:N | 10 | -23.521 | 69.513 | 20.462 | 0.001 |
| model102 | infection~geno1+S+N+geno1:S+geno1:N+S:N | 17 | -14.289 | 70.041 | 20.990 | 0.000 |
| model010 | infection~E+S+E:S | 4 | -30.998 | 70.418 | 21.367 | 0.000 |
| model104 | infection~geno1+S+N+geno1:S+S:N | 13 | -20.233 | 70.698 | 21.647 | 0.000 |
| model103 | infection~geno1+S+N+geno1:S+geno1:N | 16 | -16.131 | 70.816 | 21.765 | 0.000 |
| model201 | infection~geno2+S+N+geno2:S+geno2:N+S:N+geno2:S:N | 12 | -23.521 | 74.627 | 25.576 | 0.000 |
| model101 | infection~geno1+S+N+geno1:S+geno1:N+S:N+geno1:S:N | 21 | -14.289 | 82.424 | 33.373 | 0.000 |
| model516 | infection~geno5 | 2 | -39.807 | 83.739 | 34.688 | 0.000 |
| model513 | infection~geno5+S | 3 | -39.351 | 84.952 | 35.902 | 0.000 |
| model216 | infection~geno2 | 3 | -39.807 | 85.865 | 36.814 | 0.000 |
| model510 | infection~geno5+S+geno5:S | 4 | -39.351 | 87.123 | 38.073 | 0.000 |
| model213 | infection~geno2+S | 4 | -39.351 | 87.123 | 38.073 | 0.000 |
| model610 | infection~geno6+S+geno6:S | 4 | -40.023 | 88.467 | 39.416 | 0.000 |
| model710 | infection~geno7+S+geno7:S | 4 | -40.063 | 88.548 | 39.497 | 0.000 |
| model410 | infection~geno4+S+geno4:S | 6 | -37.841 | 88.585 | 39.534 | 0.000 |
| model019 | infection~1 | 1 | -43.967 | 89.975 | 40.924 | 0.000 |
| model310 | infection~geno3+S+geno3:S | 6 | -38.580 | 90.064 | 41.013 | 0.000 |
| model616 | infection~geno6 | 2 | -43.054 | 90.232 | 41.181 | 0.000 |
| model017 | infection~S | 2 | -43.267 | 90.658 | 41.607 | 0.000 |
| model613 | infection~geno6+S | 3 | -42.501 | 91.253 | 42.202 | 0.000 |
| model116 | infection~geno1 | 6 | -39.233 | 91.369 | 42.318 | 0.000 |
| model210 | infection~geno2+S+geno2:S | 6 | -39.351 | 91.606 | 42.555 | 0.000 |
| model716 | infection~geno7 | 2 | -43.873 | 91.870 | 42.819 | 0.000 |
| model316 | infection~geno3 | 3 | -42.887 | 92.023 | 42.973 | 0.000 |
| model416 | infection~geno4 | 3 | -43.064 | 92.379 | 43.328 | 0.000 |
| model713 | infection~geno7+S | 3 | -43.176 | 92.601 | 43.550 | 0.000 |
| model113 | infection~geno1+S | 7 | -38.763 | 92.744 | 43.693 | 0.000 |
| model413 | infection~geno4+S | 4 | -42.288 | 92.997 | 43.946 | 0.000 |
| model313 | infection~geno3+S | 4 | -42.320 | 93.062 | 44.011 | 0.000 |
| model110 | infection~geno1+S+geno1:S | 11 | -34.655 | 94.310 | 45.259 | 0.000 |

**Table S10.** Model-averaged parameter estimates are shown for the generalized linear models (with binomial errors) of bank vole infection status. The model selection table contains 117 models. The fixed factors are experiment (expt), sex (sex), number of engorged infected nymphs (N), and TLR2 genotype. TLR2 genotype was modeled in 7 different ways (geno1, geno2, geno3, geno4, geno5, geno6, and geno7). For each of the 64 parameter estimates, mean1, mean2, the lower limit (LL) and upper limit (UL) of the 95% confidence interval (CI) for mean2, and the statistical significance (p < 0.05) are shown. Mean1 is averaged over all the models that contain that parameter in the model selection table. Mean2 is averaged over a subset of models that contain that parameter. Due to problems with parameter estimation, the standard errors are very large, and none of the 95% confidence intervals overlap zero.

| Parameter | Mean1 | Mean2 | 95% LL | 95% UL | Signif |
| --- | --- | --- | --- | --- | --- |
| Intercept | -0.969 | -0.969 | -5240 | 5238 | NS |
| exptFin | 10.759 | 19.105 | -7862 | 7900 | NS |
| N | 3.982 | 3.983 | -4580 | 4588 | NS |
| sexM | 7.395 | 10.860 | -11761 | 11782 | NS |
| exptFin:N | -0.318 | -2.252 | -6392 | 6388 | NS |
| geno3C1Cy | -5.046 | -26.127 | -16236 | 16183 | NS |
| geno3CyCy | -0.792 | -4.099 | -4964 | 4956 | NS |
| geno3C1Cy:N | 8.488 | 45.986 | -13727 | 13819 | NS |
| geno3CyCy:N | 1.885 | 10.213 | -6550 | 6571 | NS |
| N:sexM | -2.234 | -8.994 | -12553 | 12535 | NS |
| exptFin:sexM | -0.045 | -0.553 | -9689 | 9688 | NS |
| geno3C1Cy:sexM | -4.298 | -56.690 | -32833 | 32719 | NS |
| geno3CyCy:sexM | -4.962 | -65.439 | -29271 | 29140 | NS |
| geno6 | -0.562 | -7.393 | -6606 | 6591 | NS |
| geno6:sexM | 0.559 | 12.000 | -8419 | 8443 | NS |
| geno6:N | 1.170 | 22.038 | -9459 | 9504 | NS |
| geno6:N:sexM | -1.713 | -54.475 | -15453 | 15344 | NS |
| geno4C3Cz | -1.539 | -21.728 | -19685 | 19641 | NS |
| geno4CzCz | 0.368 | 5.199 | -5255 | 5266 | NS |
| geno4C3Cz:sexM | 0.332 | 8.891 | -24161 | 24179 | NS |
| geno4CzCz:sexM | 2.470 | 66.232 | -25151 | 25283 | NS |
| geno4C3Cz:N | 2.188 | 35.664 | -14122 | 14194 | NS |
| geno4CzCz:N | -0.843 | -13.742 | -7501 | 7474 | NS |
| geno7 | 0.290 | 6.888 | -7769 | 7783 | NS |
| geno7:sexM | -0.284 | -10.571 | -9741 | 9720 | NS |
| geno7:N | -0.590 | -23.405 | -12067 | 12020 | NS |
| geno7:N:sexM | 0.860 | 53.600 | -16311 | 16418 | NS |
| geno5 | -0.484 | -18.416 | -11887 | 11850 | NS |
| geno3C1Cy:N:sexM | 0.008 | 2.682 | -50554 | 50560 | NS |
| geno3CyCy:N:sexM | -0.118 | -37.222 | -43830 | 43755 | NS |
| geno5:sexM | 0.006 | 1.487 | -10574 | 10577 | NS |
| geno2C2Cx | 0.006 | 1.013 | -12416 | 12418 | NS |
| geno2CxCx | -0.103 | -16.070 | -8620 | 8588 | NS |
| geno5:N | 0.039 | 5.855 | -10462 | 10474 | NS |
| geno4C3Cz:N:sexM | 0.060 | 38.469 | -29354 | 29431 | NS |
| geno4CzCz:N:sexM | 0.057 | 36.182 | -24189 | 24262 | NS |
| exptFin:N:sexM | -0.001 | -0.424 | -13160 | 13159 | NS |
| geno2C2Cx:N | 0.000 | 0.020 | -15153 | 15153 | NS |
| geno2CxCx:N | 0.001 | 2.517 | -9628 | 9633 | NS |
| geno2C2Cx:sexM | 0.000 | -0.633 | -18042 | 18041 | NS |
| geno2CxCx:sexM | 0.000 | -0.072 | -12308 | 12308 | NS |
| geno1C1C2 | 0.013 | 18.511 | -4574995 | 4575032 | NS |
| geno1C1C3 | -0.006 | -8.034 | -6425 | 6409 | NS |
| geno1C2C2 | 0.012 | 17.450 | -39530 | 39565 | NS |
| geno1C2C3 | 0.010 | 13.584 | -40431 | 40458 | NS |
| geno1C3C3 | 0.000 | 0.251 | -818 | 819 | NS |
| geno1C1C2:N | 0.000 | -0.498 | -7399411 | 7399410 | NS |
| geno1C1C3:N | 0.010 | 38.559 | -11909 | 11986 | NS |
| geno1C2C2:N | 0.000 | -0.489 | -28215 | 28214 | NS |
| geno1C2C3:N | 0.000 | NA | NA | NA | NA |
| geno1C3C3:N | 0.001 | 2.570 | -1780 | 1785 | NS |
| geno5:N:sexM | 0.000 | -1.893 | -17771 | 17767 | NS |
| geno1C1C2:sexM | -0.001 | -29.594 | -24246896 | 24246837 | NS |
| geno1C1C3:sexM | -0.001 | -33.719 | -21436 | 21368 | NS |
| geno1C2C2:sexM | -0.001 | -25.682 | -35670 | 35619 | NS |
| geno1C2C3:sexM | 0.000 | NA | NA | NA | NA |
| geno1C3C3:sexM | -0.001 | -37.748 | -18280 | 18204 | NS |
| geno2C2Cx:N:sexM | 0.000 | 0.000 | -38136 | 38136 | NS |
| geno2CxCx:N:sexM | 0.000 | 0.211 | -26508 | 26508 | NS |
| geno1C1C2:N:sexM | 0.000 | 1.278 | -97527 | 97530 | NS |
| geno1C1C3:N:sexM | 0.000 | 2.341 | -32387 | 32391 | NS |
| geno1C2C2:N:sexM | 0.000 | 1.278 | -75446 | 75448 | NS |
| geno1C2C3:N:sexM | 0.000 | NA | NA | NA | NA |
| geno1C3C3:N:sexM | 0.000 | -36.605 | -29219 | 29145 | NS |

**Table S11.** Model selection table is shown for the linear models with normal errors of bank vole infection status (infection). The analysis was done on the entire data set of 100 bank voles. Of the 117 models, the top 6 models have 99.9% of the support. The fixed factors are number of engorged *B. afzelii*-infected nymphs (N), experiment (E), sex (S), and TLR2 genotype. TLR2 genotype is modelled in 7 different ways (geno1, geno2, geno3, geno4, geno5, geno6, and geno7). For each model, the model structure, degrees of freedom (df), log likelihood, corrected AIC value (AICc), difference in AICc from the top model (Delta), and the support (Weight) are shown.

| Model ID | Model Structure | df | logLik | AICc | delta | weight |
| --- | --- | --- | --- | --- | --- | --- |
| model011 | infection~E+N+E:N | 5 | -12.287 | 35.212 | 0.000 | 44.374 |
| model007 | infection~E+S+N+E:N | 6 | -11.802 | 36.507 | 1.295 | 23.226 |
| model005 | infection~E+S+N+E:N+S:N | 7 | -11.164 | 37.546 | 2.334 | 13.817 |
| model003 | infection~E+S+N+E:S+E:N | 7 | -11.317 | 37.851 | 2.639 | 11.862 |
| model002 | infection~E+S+N+E:S+E:N+S:N | 8 | -11.032 | 39.646 | 4.434 | 4.835 |
| model001 | infection~E+S+N+E:S+E:N+S:N+E:S:N | 9 | -10.826 | 41.651 | 6.439 | 1.774 |
| model014 | infection~E+N | 4 | -21.428 | 51.277 | 16.065 | 0.014 |
| model606 | infection~geno6+S+N+geno6:S | 6 | -19.780 | 52.463 | 17.251 | 0.008 |
| model009 | infection~E+S+N | 5 | -20.939 | 52.517 | 17.305 | 0.008 |
| model008 | infection~E+S+N+S:N | 6 | -19.871 | 52.645 | 17.433 | 0.007 |
| model402 | infection~geno4+S+N+geno4:S+geno4:N+S:N | 11 | -13.990 | 52.981 | 17.769 | 0.006 |
| model604 | infection~geno6+S+N+geno6:S+S:N | 7 | -18.949 | 53.115 | 17.903 | 0.006 |
| model704 | infection~geno7+S+N+geno7:S+S:N | 7 | -19.177 | 53.572 | 18.359 | 0.005 |
| model303 | infection~geno3+S+N+geno3:S+geno3:N | 10 | -15.571 | 53.614 | 18.402 | 0.004 |
| model006 | infection~E+S+N+E:S | 6 | -20.602 | 54.107 | 18.895 | 0.004 |
| model603 | infection~geno6+S+N+geno6:S+geno6:N | 7 | -19.512 | 54.241 | 19.029 | 0.003 |
| model511 | infection~geno5+N+geno5:N | 5 | -21.875 | 54.388 | 19.176 | 0.003 |
| model403 | infection~geno4+S+N+geno4:S+geno4:N | 10 | -16.020 | 54.513 | 19.300 | 0.003 |
| model004 | infection~E+S+N+E:S+S:N | 7 | -19.858 | 54.934 | 19.722 | 0.002 |
| model706 | infection~geno7+S+N+geno7:S | 6 | -21.044 | 54.991 | 19.779 | 0.002 |
| model401 | infection~geno4+S+N+geno4:S+geno4:N+S:N+geno4:S:N | 13 | -12.445 | 55.122 | 19.910 | 0.002 |
| model302 | infection~geno3+S+N+geno3:S+geno3:N+S:N | 11 | -15.082 | 55.164 | 19.951 | 0.002 |
| model702 | infection~geno7+S+N+geno7:S+geno7:N+S:N | 8 | -18.794 | 55.171 | 19.959 | 0.002 |
| model602 | infection~geno6+S+N+geno6:S+geno6:N+S:N | 8 | -18.910 | 55.402 | 20.189 | 0.002 |
| model404 | infection~geno4+S+N+geno4:S+S:N | 9 | -17.722 | 55.445 | 20.233 | 0.002 |
| model507 | infection~geno5+S+N+geno5:N | 6 | -21.278 | 55.460 | 20.248 | 0.002 |
| model018 | infection~N | 3 | -24.743 | 55.736 | 20.524 | 0.002 |
| model012 | infection~S+N+S:N | 5 | -22.764 | 56.166 | 20.953 | 0.001 |
| model505 | infection~geno5+S+N+geno5:N+S:N | 7 | -20.530 | 56.277 | 21.065 | 0.001 |
| model411 | infection~geno4+N+geno4:N | 7 | -20.621 | 56.460 | 21.248 | 0.001 |
| model015 | infection~S+N | 4 | -24.026 | 56.473 | 21.261 | 0.001 |
| model405 | infection~geno4+S+N+geno4:N+S:N | 9 | -18.304 | 56.608 | 21.396 | 0.001 |
| model701 | infection~geno7+S+N+geno7:S+geno7:N+S:N+geno7:S:N | 9 | -18.346 | 56.693 | 21.481 | 0.001 |
| model306 | infection~geno3+S+N+geno3:S | 8 | -19.602 | 56.787 | 21.575 | 0.001 |
| model407 | infection~geno4+S+N+geno4:N | 8 | -19.628 | 56.838 | 21.626 | 0.001 |
| model406 | infection~geno4+S+N+geno4:S | 8 | -19.683 | 56.949 | 21.737 | 0.001 |
| model703 | infection~geno7+S+N+geno7:S+geno7:N | 7 | -20.967 | 57.151 | 21.939 | 0.001 |
| model614 | infection~geno6+N | 4 | -24.404 | 57.228 | 22.016 | 0.001 |
| model304 | infection~geno3+S+N+geno3:S+S:N | 9 | -18.663 | 57.327 | 22.115 | 0.001 |
| model311 | infection~geno3+N+geno3:N | 7 | -21.091 | 57.399 | 22.187 | 0.001 |
| model514 | infection~geno5+N | 4 | -24.512 | 57.445 | 22.233 | 0.001 |
| model503 | infection~geno5+S+N+geno5:S+geno5:N | 7 | -21.144 | 57.506 | 22.294 | 0.001 |
| model601 | infection~geno6+S+N+geno6:S+geno6:N+S:N+geno6:S:N | 9 | -18.791 | 57.582 | 22.370 | 0.001 |
| model307 | infection~geno3+S+N+geno3:N | 8 | -20.038 | 57.659 | 22.447 | 0.001 |
| model714 | infection~geno7+N | 4 | -24.710 | 57.841 | 22.629 | 0.001 |
| model608 | infection~geno6+S+N+S:N | 6 | -22.532 | 57.968 | 22.756 | 0.001 |
| model414 | infection~geno4+N | 5 | -23.682 | 58.002 | 22.790 | 0.000 |
| model301 | infection~geno3+S+N+geno3:S+geno3:N+S:N+geno3:S:N | 13 | -13.904 | 58.040 | 22.828 | 0.000 |
| model508 | infection~geno5+S+N+S:N | 6 | -22.591 | 58.085 | 22.873 | 0.000 |
| model609 | infection~geno6+S+N | 5 | -23.775 | 58.187 | 22.975 | 0.000 |
| model408 | infection~geno4+S+N+S:N | 7 | -21.515 | 58.248 | 23.036 | 0.000 |
| model211 | infection~geno2+N+geno2:N | 7 | -21.539 | 58.296 | 23.084 | 0.000 |
| model708 | infection~geno7+S+N+S:N | 6 | -22.745 | 58.393 | 23.181 | 0.000 |
| model509 | infection~geno5+S+N | 5 | -23.885 | 58.407 | 23.195 | 0.000 |
| model214 | infection~geno2+N | 5 | -23.887 | 58.413 | 23.201 | 0.000 |
| model611 | infection~geno6+N+geno6:N | 5 | -23.893 | 58.423 | 23.211 | 0.000 |
| model502 | infection~geno5+S+N+geno5:S+geno5:N+S:N | 8 | -20.517 | 58.616 | 23.404 | 0.000 |
| model709 | infection~geno7+S+N | 5 | -23.991 | 58.621 | 23.409 | 0.000 |
| model305 | infection~geno3+S+N+geno3:N+S:N | 9 | -19.331 | 58.662 | 23.449 | 0.000 |
| model409 | infection~geno4+S+N | 6 | -22.885 | 58.673 | 23.461 | 0.000 |
| model208 | infection~geno2+S+N+S:N | 7 | -22.019 | 59.255 | 24.043 | 0.000 |
| model314 | infection~geno3+N | 5 | -24.376 | 59.391 | 24.179 | 0.000 |
| model209 | infection~geno2+S+N | 6 | -23.244 | 59.392 | 24.180 | 0.000 |
| model607 | infection~geno6+S+N+geno6:N | 6 | -23.273 | 59.450 | 24.238 | 0.000 |
| model207 | infection~geno2+S+N+geno2:N | 8 | -20.974 | 59.531 | 24.319 | 0.000 |
| model711 | infection~geno7+N+geno7:N | 5 | -24.695 | 60.028 | 24.816 | 0.000 |
| model308 | infection~geno3+S+N+S:N | 7 | -22.429 | 60.075 | 24.863 | 0.000 |
| model605 | infection~geno6+S+N+geno6:N+S:N | 7 | -22.432 | 60.082 | 24.870 | 0.000 |
| model705 | infection~geno7+S+N+geno7:N+S:N | 7 | -22.552 | 60.322 | 25.110 | 0.000 |
| model309 | infection~geno3+S+N | 6 | -23.741 | 60.385 | 25.173 | 0.000 |
| model504 | infection~geno5+S+N+geno5:S+S:N | 7 | -22.590 | 60.398 | 25.186 | 0.000 |
| model506 | infection~geno5+S+N+geno5:S | 6 | -23.757 | 60.418 | 25.206 | 0.000 |
| model205 | infection~geno2+S+N+geno2:N+S:N | 9 | -20.211 | 60.422 | 25.210 | 0.000 |
| model501 | infection~geno5+S+N+geno5:S+geno5:N+S:N+geno5:S:N | 9 | -20.408 | 60.816 | 25.604 | 0.000 |
| model707 | infection~geno7+S+N+geno7:N | 6 | -23.960 | 60.823 | 25.611 | 0.000 |
| model111 | infection~geno1+N+geno1:N | 12 | -17.483 | 62.551 | 27.339 | 0.000 |
| model114 | infection~geno1+N | 8 | -22.502 | 62.586 | 27.374 | 0.000 |
| model109 | infection~geno1+S+N | 9 | -21.716 | 63.432 | 28.220 | 0.000 |
| model107 | infection~geno1+S+N+geno1:N | 13 | -16.653 | 63.539 | 28.326 | 0.000 |
| model108 | infection~geno1+S+N+S:N | 10 | -20.619 | 63.711 | 28.499 | 0.000 |
| model206 | infection~geno2+S+N+geno2:S | 8 | -23.184 | 63.950 | 28.738 | 0.000 |
| model204 | infection~geno2+S+N+geno2:S+S:N | 9 | -22.003 | 64.006 | 28.794 | 0.000 |
| model203 | infection~geno2+S+N+geno2:S+geno2:N | 10 | -20.835 | 64.141 | 28.929 | 0.000 |
| model103 | infection~geno1+S+N+geno1:S+geno1:N | 17 | -11.410 | 64.283 | 29.071 | 0.000 |
| model106 | infection~geno1+S+N+geno1:S | 13 | -17.278 | 64.789 | 29.577 | 0.000 |
| model105 | infection~geno1+S+N+geno1:N+S:N | 14 | -16.093 | 65.127 | 29.915 | 0.000 |
| model104 | infection~geno1+S+N+geno1:S+S:N | 14 | -16.099 | 65.139 | 29.927 | 0.000 |
| model202 | infection~geno2+S+N+geno2:S+geno2:N+S:N | 11 | -20.181 | 65.362 | 30.150 | 0.000 |
| model102 | infection~geno1+S+N+geno1:S+geno1:N+S:N | 18 | -10.810 | 66.064 | 30.851 | 0.000 |
| model016 | infection~E | 3 | -30.982 | 68.213 | 33.001 | 0.000 |
| model013 | infection~E+S | 4 | -30.637 | 69.695 | 34.483 | 0.000 |
| model201 | infection~geno2+S+N+geno2:S+geno2:N+S:N+geno2:S:N | 13 | -20.136 | 70.505 | 35.293 | 0.000 |
| model010 | infection~E+S+E:S | 5 | -30.293 | 71.223 | 36.011 | 0.000 |
| model101 | infection~geno1+S+N+geno1:S+geno1:N+S:N+geno1:S:N | 22 | -9.519 | 76.181 | 40.969 | 0.000 |
| model516 | infection~geno5 | 3 | -39.221 | 84.692 | 49.480 | 0.000 |
| model610 | infection~geno6+S+geno6:S | 5 | -37.123 | 84.884 | 49.671 | 0.000 |
| model513 | infection~geno5+S | 4 | -38.794 | 86.009 | 50.797 | 0.000 |
| model216 | infection~geno2 | 4 | -38.949 | 86.319 | 51.107 | 0.000 |
| model710 | infection~geno7+S+geno7:S | 5 | -37.947 | 86.533 | 51.321 | 0.000 |
| model019 | infection~1 | 2 | -41.547 | 87.218 | 52.006 | 0.000 |
| model616 | infection~geno6 | 3 | -40.618 | 87.487 | 52.275 | 0.000 |
| model213 | infection~geno2+S | 5 | -38.489 | 87.616 | 52.404 | 0.000 |
| model510 | infection~geno5+S+geno5:S | 5 | -38.645 | 87.929 | 52.717 | 0.000 |
| model017 | infection~S | 3 | -40.848 | 87.945 | 52.733 | 0.000 |
| model310 | infection~geno3+S+geno3:S | 7 | -36.463 | 88.144 | 52.932 | 0.000 |
| model613 | infection~geno6+S | 4 | -40.064 | 88.550 | 53.338 | 0.000 |
| model716 | infection~geno7 | 3 | -41.452 | 89.154 | 53.942 | 0.000 |
| model410 | infection~geno4+S+geno4:S | 7 | -37.014 | 89.246 | 54.034 | 0.000 |
| model316 | infection~geno3 | 4 | -40.505 | 89.431 | 54.219 | 0.000 |
| model416 | infection~geno4 | 4 | -40.565 | 89.551 | 54.339 | 0.000 |
| model713 | infection~geno7+S | 4 | -40.755 | 89.931 | 54.719 | 0.000 |
| model413 | infection~geno4+S | 5 | -39.789 | 90.217 | 55.005 | 0.000 |
| model313 | infection~geno3+S | 5 | -39.939 | 90.517 | 55.305 | 0.000 |
| model116 | infection~geno1 | 7 | -38.231 | 91.680 | 56.468 | 0.000 |
| model210 | infection~geno2+S+geno2:S | 7 | -38.374 | 91.966 | 56.754 | 0.000 |
| model113 | infection~geno1+S | 8 | -37.755 | 93.092 | 57.879 | 0.000 |
| model110 | infection~geno1+S+geno1:S | 12 | -33.611 | 94.809 | 59.597 | 0.000 |

**Table S12.** Model-averaged parameter estimates are shown for the linear models (with normal errors) of bank vole infection status. The model selection table contains 117 models. The fixed factors are experiment (expt), sex (sex), number of engorged infected nymphs (N), and TLR2 genotype. TLR2 genotype was modeled in 7 different ways (geno1, geno2, geno3, geno4, geno5, geno6, and geno7). For each of the 64 parameter estimates, mean1, mean2, the lower limit (LL) and upper limit (UL) of the 95% confidence interval (CI) for mean2, and the statistical significance (p < 0.05) are shown. Mean1 is averaged over all the models that contain that parameter in the model selection table. Mean2 is averaged over a subset of models that contain that parameter.

| Parameter | Mean1 | Mean2 | 95% LL | 95% UL | Signif |
| --- | --- | --- | --- | --- | --- |
| **Intercept** | **0.350** | **0.350** | **0.203** | **0.497** | **P<0.05** |
| **exptFin** | **0.625** | **0.626** | **0.374** | **0.878** | **P<0.05** |
| **N** | **0.298** | **0.298** | **0.201** | **0.395** | **P<0.05** |
| **exptFin:N** | **-0.290** | **-0.290** | **-0.425** | **-0.156** | **P<0.05** |
| sexM | 0.057 | 0.103 | -0.085 | 0.292 | NS |
| N:sexM | -0.013 | -0.063 | -0.193 | 0.066 | NS |
| exptFin:sexM | -0.019 | -0.105 | -0.383 | 0.174 | NS |
| exptFin:N:sexM | 0.002 | 0.085 | -0.189 | 0.358 | NS |
| geno6 | 0.000 | 0.125 | -0.012 | 0.263 | NS |
| **geno6:sexM** | **0.000** | **-0.204** | **-0.360** | **-0.049** | **P<0.05** |
| geno4C3Cz | 0.000 | -0.375 | -0.754 | 0.004 | NS |
| geno4CzCz | 0.000 | -0.141 | -0.478 | 0.195 | NS |
| geno4C3Cz:sexM | 0.000 | 0.207 | -0.169 | 0.584 | NS |
| **geno4CzCz:sexM** | **0.000** | **0.436** | **0.031** | **0.841** | **P<0.05** |
| geno4C3Cz:N | 0.000 | 0.156 | -0.028 | 0.341 | NS |
| geno4CzCz:N | 0.000 | -0.020 | -0.208 | 0.167 | NS |
| geno7 | 0.000 | -0.078 | -0.220 | 0.063 | NS |
| **geno7:sexM** | **0.000** | **0.196** | **0.023** | **0.368** | **P<0.05** |
| geno3C1Cy | 0.000 | -0.106 | -0.468 | 0.256 | NS |
| geno3CyCy | 0.000 | 0.252 | -0.073 | 0.576 | NS |
| geno3C1Cy:sexM | 0.000 | -0.252 | -0.621 | 0.117 | NS |
| **geno3CyCy:sexM** | **0.000** | **-0.442** | **-0.791** | **-0.093** | **P<0.05** |
| geno3C1Cy:N | 0.000 | 0.172 | -0.010 | 0.353 | NS |
| geno3CyCy:N | 0.000 | -0.023 | -0.194 | 0.148 | NS |
| geno6:N | 0.000 | -0.025 | -0.112 | 0.062 | NS |
| geno5 | 0.000 | -0.246 | -0.576 | 0.085 | NS |
| **geno5:N** | **0.000** | **0.130** | **0.007** | **0.253** | **P<0.05** |
| geno4C3Cz:N:sexM | 0.000 | 0.018 | -0.321 | 0.357 | NS |
| geno4CzCz:N:sexM | 0.000 | -0.207 | -0.525 | 0.112 | NS |
| geno7:N | 0.000 | -0.017 | -0.114 | 0.080 | NS |
| geno7:N:sexM | 0.000 | -0.073 | -0.231 | 0.086 | NS |
| geno5:sexM | 0.000 | 0.045 | -0.242 | 0.331 | NS |
| geno6:N:sexM | 0.000 | 0.040 | -0.129 | 0.208 | NS |
| geno3C1Cy:N:sexM | 0.000 | 0.239 | -0.121 | 0.599 | NS |
| geno3CyCy:N:sexM | 0.000 | 0.217 | -0.126 | 0.559 | NS |
| geno2C2Cx | 0.000 | 0.057 | -0.544 | 0.658 | NS |
| geno2CxCx | 0.000 | -0.247 | -0.875 | 0.381 | NS |
| geno2C2Cx:N | 0.000 | 0.000 | -0.403 | 0.403 | NS |
| geno2CxCx:N | 0.000 | 0.219 | -0.052 | 0.489 | NS |
| geno5:N:sexM | 0.000 | -0.072 | -0.393 | 0.249 | NS |
| geno1C1C2 | 0.000 | 0.320 | -0.308 | 0.949 | NS |
| geno1C1C3 | 0.000 | -0.139 | -0.489 | 0.210 | NS |
| geno1C2C2 | 0.000 | 0.251 | -0.372 | 0.875 | NS |
| geno1C2C3 | 0.000 | -0.143 | -0.804 | 0.518 | NS |
| geno1C3C3 | 0.000 | 0.126 | -0.166 | 0.418 | NS |
| geno1C1C2:N | 0.000 | -0.161 | -0.625 | 0.303 | NS |
| geno1C1C3:N | 0.000 | 0.176 | -0.005 | 0.357 | NS |
| geno1C2C2:N | 0.000 | -0.168 | -0.457 | 0.120 | NS |
| geno1C2C3:N | 0.000 | NA | NA | NA | NA |
| geno1C3C3:N | 0.000 | -0.015 | -0.191 | 0.160 | NS |
| geno2C2Cx:sexM | 0.000 | -0.018 | -0.635 | 0.600 | NS |
| geno2CxCx:sexM | 0.000 | 0.033 | -0.430 | 0.496 | NS |
| geno1C1C2:sexM | 0.000 | -0.276 | -0.802 | 0.251 | NS |
| geno1C1C3:sexM | 0.000 | -0.252 | -0.594 | 0.090 | NS |
| geno1C2C2:sexM | 0.000 | -0.291 | -0.741 | 0.160 | NS |
| geno1C2C3:sexM | 0.000 | NA | NA | NA | NA |
| **geno1C3C3:sexM** | **0.000** | **-0.474** | **-0.798** | **-0.151** | **P<0.05** |
| geno2C2Cx:N:sexM | 0.000 | 0.000 | -1.119 | 1.119 | NS |
| geno2CxCx:N:sexM | 0.000 | -0.082 | -0.873 | 0.710 | NS |
| geno1C1C2:N:sexM | 0.000 | 0.233 | -0.777 | 1.244 | NS |
| geno1C1C3:N:sexM | 0.000 | 0.256 | -0.114 | 0.626 | NS |
| geno1C2C2:N:sexM | 0.000 | 0.233 | -0.555 | 1.021 | NS |
| geno1C2C3:N:sexM | 0.000 | NA | NA | NA | NA |
| geno1C3C3:N:sexM | 0.000 | 0.190 | -0.185 | 0.565 | NS |

**Table S13.** Model selection table is shown for the linear mixed effects models of the *B. afzelii* spirochete load in bank vole tissues. Spirochete load is standardized per mg of DNA and was log10-transformed to normalize the residuals (log10(spiro.per.mg.DNA)). The analysis was done on the subset of 84 bank voles infected with *B. afzelii*. Of the 117 models, the top 5 models have 99.4% of the support. The fixed factors are experiment (E), organ (O), sex (S), and TLR2 genotype. TLR2 genotype is modelled in 7 different ways (geno1, geno2, geno3, geno4, geno5, geno6, and geno7). For each model, the model structure, degrees of freedom (df), log likelihood, corrected AIC value (AICc), difference in AICc from the top model (Delta), and the support (Weight) are shown.

| Model | Model Structure | df | logLik | AICc | delta | weight |
| --- | --- | --- | --- | --- | --- | --- |
| model014 | log10(spiro.per.mg.DNA)~E+O | 7 | -408.954 | 832.250 | 0.000 | 76.959 |
| model011 | log10(spiro.per.mg.DNA)~E+O+E:O | 10 | -407.676 | 836.029 | 3.779 | 11.632 |
| model009 | log10(spiro.per.mg.DNA)~E+S+O | 8 | -410.178 | 836.796 | 4.547 | 7.925 |
| model006 | log10(spiro.per.mg.DNA)~E+S+O+E:S | 9 | -410.639 | 839.830 | 7.580 | 1.739 |
| model007 | log10(spiro.per.mg.DNA)~E+S+O+E:O | 11 | -408.900 | 840.614 | 8.364 | 1.175 |
| model003 | log10(spiro.per.mg.DNA)~E+S+O+E:S+E:O | 12 | -409.361 | 843.688 | 11.438 | 0.253 |
| model008 | log10(spiro.per.mg.DNA)~E+S+O+S:O | 11 | -410.562 | 843.939 | 11.689 | 0.223 |
| model004 | log10(spiro.per.mg.DNA)~E+S+O+E:S+S:O | 12 | -411.023 | 847.012 | 14.762 | 0.048 |
| model005 | log10(spiro.per.mg.DNA)~E+S+O+E:O+S:O | 14 | -409.259 | 847.826 | 15.576 | 0.032 |
| model002 | log10(spiro.per.mg.DNA)~E+S+O+E:S+E:O+S:O | 15 | -409.720 | 850.939 | 18.690 | 0.007 |
| model018 | log10(spiro.per.mg.DNA)~O | 6 | -419.902 | 852.059 | 19.809 | 0.004 |
| model514 | log10(spiro.per.mg.DNA)~geno5+O | 7 | -420.093 | 854.527 | 22.277 | 0.001 |
| model001 | log10(spiro.per.mg.DNA)~E+S+O+E:S+E:O+S:O+E:S:O | 18 | -408.398 | 854.955 | 22.705 | 0.001 |
| model015 | log10(spiro.per.mg.DNA)~S+O | 7 | -420.954 | 856.250 | 24.001 | 0.000 |
| model214 | log10(spiro.per.mg.DNA)~geno2+O | 8 | -419.992 | 856.424 | 24.174 | 0.000 |
| model714 | log10(spiro.per.mg.DNA)~geno7+O | 7 | -421.123 | 856.588 | 24.338 | 0.000 |
| model614 | log10(spiro.per.mg.DNA)~geno6+O | 7 | -421.364 | 857.069 | 24.819 | 0.000 |
| model414 | log10(spiro.per.mg.DNA)~geno4+O | 8 | -420.500 | 857.441 | 25.191 | 0.000 |
| model314 | log10(spiro.per.mg.DNA)~geno3+O | 8 | -421.045 | 858.530 | 26.280 | 0.000 |
| model509 | log10(spiro.per.mg.DNA)~geno5+S+O | 8 | -421.178 | 858.797 | 26.547 | 0.000 |
| model411 | log10(spiro.per.mg.DNA)~geno4+O+geno4:O | 14 | -415.171 | 859.651 | 27.402 | 0.000 |
| model209 | log10(spiro.per.mg.DNA)~geno2+S+O | 9 | -421.070 | 860.692 | 28.442 | 0.000 |
| model709 | log10(spiro.per.mg.DNA)~geno7+S+O | 8 | -422.189 | 860.818 | 28.569 | 0.000 |
| model609 | log10(spiro.per.mg.DNA)~geno6+S+O | 8 | -422.412 | 861.264 | 29.014 | 0.000 |
| model311 | log10(spiro.per.mg.DNA)~geno3+O+geno3:O | 14 | -416.101 | 861.510 | 29.260 | 0.000 |
| model409 | log10(spiro.per.mg.DNA)~geno4+S+O | 9 | -421.559 | 861.670 | 29.420 | 0.000 |
| model506 | log10(spiro.per.mg.DNA)~geno5+S+O+geno5:S | 9 | -421.879 | 862.310 | 30.060 | 0.000 |
| model309 | log10(spiro.per.mg.DNA)~geno3+S+O | 9 | -422.099 | 862.751 | 30.501 | 0.000 |
| model012 | log10(spiro.per.mg.DNA)~S+O+S:O | 10 | -421.339 | 863.354 | 31.104 | 0.000 |
| model114 | log10(spiro.per.mg.DNA)~geno1+O | 11 | -420.349 | 863.513 | 31.263 | 0.000 |
| model611 | log10(spiro.per.mg.DNA)~geno6+O+geno6:O | 10 | -421.544 | 863.764 | 31.514 | 0.000 |
| model706 | log10(spiro.per.mg.DNA)~geno7+S+O+geno7:S | 9 | -422.635 | 863.823 | 31.573 | 0.000 |
| model407 | log10(spiro.per.mg.DNA)~geno4+S+O+geno4:O | 15 | -416.230 | 863.960 | 31.711 | 0.000 |
| model606 | log10(spiro.per.mg.DNA)~geno6+S+O+geno6:S | 9 | -422.844 | 864.239 | 31.990 | 0.000 |
| model206 | log10(spiro.per.mg.DNA)~geno2+S+O+geno2:S | 11 | -420.902 | 864.618 | 32.369 | 0.000 |
| model711 | log10(spiro.per.mg.DNA)~geno7+O+geno7:O | 10 | -422.354 | 865.386 | 33.136 | 0.000 |
| model307 | log10(spiro.per.mg.DNA)~geno3+S+O+geno3:O | 15 | -417.156 | 865.811 | 33.561 | 0.000 |
| model406 | log10(spiro.per.mg.DNA)~geno4+S+O+geno4:S | 11 | -421.500 | 865.816 | 33.566 | 0.000 |
| model508 | log10(spiro.per.mg.DNA)~geno5+S+O+S:O | 11 | -421.562 | 865.939 | 33.690 | 0.000 |
| model306 | log10(spiro.per.mg.DNA)~geno3+S+O+geno3:S | 11 | -421.645 | 866.104 | 33.855 | 0.000 |
| model511 | log10(spiro.per.mg.DNA)~geno5+O+geno5:O | 10 | -422.779 | 866.235 | 33.985 | 0.000 |
| model109 | log10(spiro.per.mg.DNA)~geno1+S+O | 12 | -421.412 | 867.789 | 35.540 | 0.000 |
| model208 | log10(spiro.per.mg.DNA)~geno2+S+O+S:O | 12 | -421.454 | 867.874 | 35.624 | 0.000 |
| model708 | log10(spiro.per.mg.DNA)~geno7+S+O+S:O | 11 | -422.573 | 867.961 | 35.711 | 0.000 |
| model607 | log10(spiro.per.mg.DNA)~geno6+S+O+geno6:O | 11 | -422.592 | 867.998 | 35.749 | 0.000 |
| model403 | log10(spiro.per.mg.DNA)~geno4+S+O+geno4:S+geno4:O | 17 | -416.172 | 868.268 | 36.018 | 0.000 |
| model608 | log10(spiro.per.mg.DNA)~geno6+S+O+S:O | 11 | -422.796 | 868.407 | 36.157 | 0.000 |
| model408 | log10(spiro.per.mg.DNA)~geno4+S+O+S:O | 12 | -421.943 | 868.852 | 36.602 | 0.000 |
| model303 | log10(spiro.per.mg.DNA)~geno3+S+O+geno3:S+geno3:O | 17 | -416.701 | 869.326 | 37.077 | 0.000 |
| model504 | log10(spiro.per.mg.DNA)~geno5+S+O+geno5:S+S:O | 12 | -422.263 | 869.492 | 37.242 | 0.000 |
| model707 | log10(spiro.per.mg.DNA)~geno7+S+O+geno7:O | 11 | -423.420 | 869.655 | 37.405 | 0.000 |
| model308 | log10(spiro.per.mg.DNA)~geno3+S+O+S:O | 12 | -422.483 | 869.933 | 37.683 | 0.000 |
| model211 | log10(spiro.per.mg.DNA)~geno2+O+geno2:O | 14 | -420.581 | 870.470 | 38.220 | 0.000 |
| model507 | log10(spiro.per.mg.DNA)~geno5+S+O+geno5:O | 11 | -423.865 | 870.544 | 38.294 | 0.000 |
| model704 | log10(spiro.per.mg.DNA)~geno7+S+O+geno7:S+S:O | 12 | -423.020 | 871.005 | 38.755 | 0.000 |
| model405 | log10(spiro.per.mg.DNA)~geno4+S+O+geno4:O+S:O | 18 | -416.426 | 871.010 | 38.760 | 0.000 |
| model603 | log10(spiro.per.mg.DNA)~geno6+S+O+geno6:S+geno6:O | 12 | -423.024 | 871.013 | 38.763 | 0.000 |
| model604 | log10(spiro.per.mg.DNA)~geno6+S+O+geno6:S+S:O | 12 | -423.228 | 871.421 | 39.172 | 0.000 |
| model204 | log10(spiro.per.mg.DNA)~geno2+S+O+geno2:S+S:O | 14 | -421.286 | 871.880 | 39.630 | 0.000 |
| model703 | log10(spiro.per.mg.DNA)~geno7+S+O+geno7:S+geno7:O | 12 | -423.867 | 872.699 | 40.450 | 0.000 |
| model404 | log10(spiro.per.mg.DNA)~geno4+S+O+geno4:S+S:O | 14 | -421.884 | 873.077 | 40.827 | 0.000 |
| model304 | log10(spiro.per.mg.DNA)~geno3+S+O+geno3:S+S:O | 14 | -422.029 | 873.366 | 41.116 | 0.000 |
| model305 | log10(spiro.per.mg.DNA)~geno3+S+O+geno3:O+S:O | 18 | -417.623 | 873.405 | 41.155 | 0.000 |
| model503 | log10(spiro.per.mg.DNA)~geno5+S+O+geno5:S+geno5:O | 12 | -424.565 | 874.096 | 41.847 | 0.000 |
| model106 | log10(spiro.per.mg.DNA)~geno1+S+O+geno1:S | 16 | -420.390 | 874.485 | 42.235 | 0.000 |
| model207 | log10(spiro.per.mg.DNA)~geno2+S+O+geno2:O | 15 | -421.659 | 874.818 | 42.568 | 0.000 |
| model108 | log10(spiro.per.mg.DNA)~geno1+S+O+S:O | 15 | -421.796 | 875.092 | 42.842 | 0.000 |
| model605 | log10(spiro.per.mg.DNA)~geno6+S+O+geno6:O+S:O | 14 | -422.973 | 875.254 | 43.004 | 0.000 |
| model402 | log10(spiro.per.mg.DNA)~geno4+S+O+geno4:S+geno4:O+S:O | 20 | -416.368 | 875.402 | 43.152 | 0.000 |
| model705 | log10(spiro.per.mg.DNA)~geno7+S+O+geno7:O+S:O | 14 | -423.785 | 876.879 | 44.629 | 0.000 |
| model302 | log10(spiro.per.mg.DNA)~geno3+S+O+geno3:S+geno3:O+S:O | 20 | -417.169 | 877.004 | 44.754 | 0.000 |
| model111 | log10(spiro.per.mg.DNA)~geno1+O+geno1:O | 26 | -410.264 | 877.071 | 44.821 | 0.000 |
| model505 | log10(spiro.per.mg.DNA)~geno5+S+O+geno5:O+S:O | 14 | -424.355 | 878.018 | 45.768 | 0.000 |
| model602 | log10(spiro.per.mg.DNA)~geno6+S+O+geno6:S+geno6:O+S:O | 15 | -423.404 | 878.309 | 46.059 | 0.000 |
| model203 | log10(spiro.per.mg.DNA)~geno2+S+O+geno2:S+geno2:O | 17 | -421.491 | 878.906 | 46.656 | 0.000 |
| model702 | log10(spiro.per.mg.DNA)~geno7+S+O+geno7:S+geno7:O+S:O | 15 | -424.232 | 879.963 | 47.714 | 0.000 |
| model107 | log10(spiro.per.mg.DNA)~geno1+S+O+geno1:O | 27 | -411.326 | 881.562 | 49.312 | 0.000 |
| model502 | log10(spiro.per.mg.DNA)~geno5+S+O+geno5:S+geno5:O+S:O | 15 | -425.056 | 881.611 | 49.361 | 0.000 |
| model301 | log10(spiro.per.mg.DNA)~geno3+S+O+geno3:S+geno3:O+S:O+geno3:S:O | 26 | -412.656 | 881.856 | 49.607 | 0.000 |
| model104 | log10(spiro.per.mg.DNA)~geno1+S+O+geno1:S+S:O | 19 | -420.774 | 881.953 | 49.703 | 0.000 |
| model205 | log10(spiro.per.mg.DNA)~geno2+S+O+geno2:O+S:O | 18 | -422.147 | 882.452 | 50.202 | 0.000 |
| model401 | log10(spiro.per.mg.DNA)~geno4+S+O+geno4:S+geno4:O+S:O+geno4:S:O | 26 | -413.093 | 882.731 | 50.481 | 0.000 |
| model601 | log10(spiro.per.mg.DNA)~geno6+S+O+geno6:S+geno6:O+S:O+geno6:S:O | 18 | -423.670 | 885.498 | 53.248 | 0.000 |
| model202 | log10(spiro.per.mg.DNA)~geno2+S+O+geno2:S+geno2:O+S:O | 20 | -421.979 | 886.624 | 54.374 | 0.000 |
| model501 | log10(spiro.per.mg.DNA)~geno5+S+O+geno5:S+geno5:O+S:O+geno5:S:O | 18 | -424.965 | 888.088 | 55.839 | 0.000 |
| model701 | log10(spiro.per.mg.DNA)~geno7+S+O+geno7:S+geno7:O+S:O+geno7:S:O | 18 | -425.387 | 888.932 | 56.682 | 0.000 |
| model103 | log10(spiro.per.mg.DNA)~geno1+S+O+geno1:S+geno1:O | 31 | -410.304 | 889.135 | 56.885 | 0.000 |
| model105 | log10(spiro.per.mg.DNA)~geno1+S+O+geno1:O+S:O | 30 | -411.840 | 889.778 | 57.529 | 0.000 |
| model201 | log10(spiro.per.mg.DNA)~geno2+S+O+geno2:S+geno2:O+S:O+geno2:S:O | 26 | -417.129 | 890.801 | 58.551 | 0.000 |
| model102 | log10(spiro.per.mg.DNA)~geno1+S+O+geno1:S+geno1:O+S:O | 34 | -410.818 | 897.543 | 65.293 | 0.000 |
| model016 | log10(spiro.per.mg.DNA)~E | 4 | -448.869 | 905.860 | 73.610 | 0.000 |
| model101 | log10(spiro.per.mg.DNA)~geno1+S+O+geno1:S+geno1:O+S:O+geno1:S:O | 46 | -400.595 | 908.151 | 75.902 | 0.000 |
| model013 | log10(spiro.per.mg.DNA)~E+S | 5 | -450.093 | 910.368 | 78.119 | 0.000 |
| model010 | log10(spiro.per.mg.DNA)~E+S+E:S | 6 | -450.554 | 913.364 | 81.114 | 0.000 |
| model019 | log10(spiro.per.mg.DNA)~1 | 3 | -459.817 | 925.707 | 93.457 | 0.000 |
| model516 | log10(spiro.per.mg.DNA)~geno5 | 4 | -460.008 | 928.137 | 95.887 | 0.000 |
| model017 | log10(spiro.per.mg.DNA)~S | 4 | -460.870 | 929.860 | 97.611 | 0.000 |
| model216 | log10(spiro.per.mg.DNA)~geno2 | 5 | -459.907 | 929.996 | 97.746 | 0.000 |
| model716 | log10(spiro.per.mg.DNA)~geno7 | 4 | -461.039 | 930.198 | 97.948 | 0.000 |
| model616 | log10(spiro.per.mg.DNA)~geno6 | 4 | -461.279 | 930.679 | 98.429 | 0.000 |
| model416 | log10(spiro.per.mg.DNA)~geno4 | 5 | -460.415 | 931.013 | 98.763 | 0.000 |
| model316 | log10(spiro.per.mg.DNA)~geno3 | 5 | -460.960 | 932.102 | 99.852 | 0.000 |
| model513 | log10(spiro.per.mg.DNA)~geno5+S | 5 | -461.093 | 932.369 | 100.119 | 0.000 |
| model213 | log10(spiro.per.mg.DNA)~geno2+S | 6 | -460.985 | 934.226 | 101.976 | 0.000 |
| model713 | log10(spiro.per.mg.DNA)~geno7+S | 5 | -462.104 | 934.390 | 102.141 | 0.000 |
| model613 | log10(spiro.per.mg.DNA)~geno6+S | 5 | -462.327 | 934.836 | 102.586 | 0.000 |
| model413 | log10(spiro.per.mg.DNA)~geno4+S | 6 | -461.474 | 935.204 | 102.954 | 0.000 |
| model510 | log10(spiro.per.mg.DNA)~geno5+S+geno5:S | 6 | -461.794 | 935.843 | 103.594 | 0.000 |
| model313 | log10(spiro.per.mg.DNA)~geno3+S | 6 | -462.015 | 936.285 | 104.035 | 0.000 |
| model116 | log10(spiro.per.mg.DNA)~geno1 | 8 | -460.264 | 936.969 | 104.719 | 0.000 |
| model710 | log10(spiro.per.mg.DNA)~geno7+S+geno7:S | 6 | -462.551 | 937.357 | 105.107 | 0.000 |
| model610 | log10(spiro.per.mg.DNA)~geno6+S+geno6:S | 6 | -462.759 | 937.773 | 105.523 | 0.000 |
| model210 | log10(spiro.per.mg.DNA)~geno2+S+geno2:S | 8 | -460.817 | 938.074 | 105.825 | 0.000 |
| model410 | log10(spiro.per.mg.DNA)~geno4+S+geno4:S | 8 | -461.416 | 939.272 | 107.022 | 0.000 |
| model310 | log10(spiro.per.mg.DNA)~geno3+S+geno3:S | 8 | -461.560 | 939.560 | 107.311 | 0.000 |
| model113 | log10(spiro.per.mg.DNA)~geno1+S | 9 | -461.327 | 941.206 | 108.956 | 0.000 |
| model110 | log10(spiro.per.mg.DNA)~geno1+S+geno1:S | 13 | -460.305 | 947.740 | 115.491 | 0.000 |

**Table S14.** Model-averaged parameter estimates are shown for the linear mixed effects models (with normal errors) of spirochete loads in the bank vole tissues. The spirochete loads were standardized per mg of DNA before being log10-transformed. The model selection table contains 117 models. The fixed factors are experiment (expt), sex (sex), organ (org), and TLR2 genotype. TLR2 genotype was modelled in 7 different ways (geno1, geno2, geno3, geno4, geno5, geno6, and geno7). For each of the 124 parameter estimates, mean1, mean2, the lower limit (LL) and upper limit (UL) of the 95% confidence interval (CI) for mean2, and the statistical significance (p < 0.05) are shown. Mean1 is averaged over all the models that contain that parameter in the model selection table. Mean2 is averaged over a subset of models that contain that parameter.

| Parameter | Mean1 | Mean2 | 95% LL | 95% UL | Signif |
| --- | --- | --- | --- | --- | --- |
| **Intercept** | **2.613** | **2.613** | **2.358** | **2.868** | **P<0.05** |
| **exptFin** | **0.596** | **0.596** | **0.297** | **0.895** | **P<0.05** |
| **orgEar** | **0.913** | **0.913** | **0.586** | **1.240** | **P<0.05** |
| **orgJoint** | **0.981** | **0.981** | **0.733** | **1.229** | **P<0.05** |
| **orgSkin** | **0.846** | **0.846** | **0.581** | **1.110** | **P<0.05** |
| **exptFin:orgEar** | **0.073** | **0.554** | **0.104** | **1.005** | **P<0.05** |
| exptFin:orgJoint | 0.015 | 0.111 | -0.339 | 0.562 | NS |
| exptFin:orgSkin | 0.034 | 0.263 | -0.188 | 0.713 | NS |
| sexM | -0.002 | -0.015 | -0.288 | 0.258 | NS |
| exptFin:sexM | 0.001 | 0.073 | -0.401 | 0.547 | NS |
| orgEar:sexM | 0.000 | 0.118 | -0.331 | 0.566 | NS |
| orgJoint:sexM | 0.001 | 0.389 | -0.059 | 0.838 | NS |
| orgSkin:sexM | 0.001 | 0.251 | -0.198 | 0.699 | NS |
| geno5 | 0.000 | -0.145 | -0.325 | 0.035 | NS |
| exptFin:orgEar:sexM | 0.000 | 0.361 | -0.543 | 1.265 | NS |
| exptFin:orgJoint:sexM | 0.000 | 0.665 | -0.239 | 1.569 | NS |
| exptFin:orgSkin:sexM | 0.000 | 0.576 | -0.328 | 1.480 | NS |
| geno2C2Cx | 0.000 | -0.148 | -0.687 | 0.391 | NS |
| geno2CxCx | 0.000 | -0.293 | -0.679 | 0.093 | NS |
| geno7 | 0.000 | 0.068 | -0.091 | 0.226 | NS |
| geno6 | 0.000 | 0.043 | -0.126 | 0.212 | NS |
| geno4C3Cz | 0.000 | -0.175 | -0.601 | 0.251 | NS |
| geno4CzCz | 0.000 | 0.073 | -0.292 | 0.439 | NS |
| geno3C1Cy | 0.000 | -0.111 | -0.517 | 0.295 | NS |
| geno3CyCy | 0.000 | 0.065 | -0.299 | 0.429 | NS |
| geno4C3Cz:orgEar | 0.000 | 0.322 | -0.301 | 0.944 | NS |
| geno4CzCz:orgEar | 0.000 | 0.151 | -0.360 | 0.662 | NS |
| **geno4C3Cz:orgJoint** | **0.000** | **-0.630** | **-1.252** | **-0.007** | **P<0.05** |
| geno4CzCz:orgJoint | 0.000 | -0.136 | -0.647 | 0.375 | NS |
| geno4C3Cz:orgSkin | 0.000 | 0.521 | -0.102 | 1.143 | NS |
| geno4CzCz:orgSkin | 0.000 | 0.501 | -0.010 | 1.012 | NS |
| geno3C1Cy:orgEar | 0.000 | 0.250 | -0.334 | 0.835 | NS |
| geno3CyCy:orgEar | 0.000 | 0.102 | -0.436 | 0.640 | NS |
| geno3C1Cy:orgJoint | 0.000 | -0.446 | -1.030 | 0.138 | NS |
| geno3CyCy:orgJoint | 0.000 | 0.186 | -0.352 | 0.724 | NS |
| geno3C1Cy:orgSkin | 0.000 | -0.278 | -0.862 | 0.306 | NS |
| geno3CyCy:orgSkin | 0.000 | -0.432 | -0.970 | 0.106 | NS |
| geno5:sexM | 0.000 | 0.056 | -0.319 | 0.430 | NS |
| geno1C1C2 | 0.000 | 0.048 | -0.456 | 0.553 | NS |
| geno1C1C3 | 0.000 | -0.223 | -0.619 | 0.172 | NS |
| geno1C2C2 | 0.000 | 0.219 | -0.221 | 0.658 | NS |
| geno1C2C3 | 0.000 | 0.254 | -0.992 | 1.500 | NS |
| geno1C3C3 | 0.000 | -0.035 | -0.396 | 0.326 | NS |
| geno6:orgEar | 0.000 | 0.035 | -0.235 | 0.305 | NS |
| geno6:orgJoint | 0.000 | 0.136 | -0.134 | 0.406 | NS |
| geno6:orgSkin | 0.000 | -0.211 | -0.481 | 0.059 | NS |
| geno7:sexM | 0.000 | 0.155 | -0.154 | 0.464 | NS |
| geno6:sexM | 0.000 | -0.157 | -0.483 | 0.170 | NS |
| geno2C2Cx:sexM | 0.000 | 0.061 | -1.053 | 1.176 | NS |
| geno2CxCx:sexM | 0.000 | 0.113 | -0.699 | 0.926 | NS |
| geno7:orgEar | 0.000 | 0.066 | -0.190 | 0.323 | NS |
| geno7:orgJoint | 0.000 | -0.020 | -0.277 | 0.237 | NS |
| geno7:orgSkin | 0.000 | 0.241 | -0.016 | 0.497 | NS |
| geno4C3Cz:sexM | 0.000 | -0.021 | -0.786 | 0.744 | NS |
| geno4CzCz:sexM | 0.000 | 0.255 | -0.374 | 0.883 | NS |
| geno3C1Cy:sexM | 0.000 | -0.474 | -1.190 | 0.241 | NS |
| geno3CyCy:sexM | 0.000 | -0.358 | -1.018 | 0.302 | NS |
| geno5:orgEar | 0.000 | -0.133 | -0.434 | 0.169 | NS |
| geno5:orgJoint | 0.000 | -0.138 | -0.439 | 0.163 | NS |
| geno5:orgSkin | 0.000 | -0.069 | -0.371 | 0.232 | NS |
| geno2C2Cx:orgEar | 0.000 | -0.101 | -1.009 | 0.807 | NS |
| geno2CxCx:orgEar | 0.000 | -0.254 | -0.902 | 0.395 | NS |
| geno2C2Cx:orgJoint | 0.000 | -0.324 | -1.231 | 0.584 | NS |
| geno2CxCx:orgJoint | 0.000 | -0.297 | -0.945 | 0.351 | NS |
| geno2C2Cx:orgSkin | 0.000 | -0.392 | -1.300 | 0.516 | NS |
| geno2CxCx:orgSkin | 0.000 | -0.193 | -0.841 | 0.455 | NS |
| geno1C1C2:sexM | 0.000 | -0.400 | -1.425 | 0.625 | NS |
| geno1C1C3:sexM | 0.000 | -0.500 | -1.303 | 0.303 | NS |
| geno1C2C2:sexM | 0.000 | -0.413 | -1.339 | 0.514 | NS |
| geno1C3C3:sexM | 0.000 | -0.423 | -1.158 | 0.312 | NS |
| geno1C1C2:orgEar | 0.000 | 0.181 | -0.648 | 1.011 | NS |
| geno1C1C3:orgEar | 0.000 | 0.283 | -0.366 | 0.931 | NS |
| geno1C2C2:orgEar | 0.000 | 0.320 | -0.400 | 1.041 | NS |
| geno1C2C3:orgEar | 0.000 | 0.525 | -1.529 | 2.578 | NS |
| geno1C3C3:orgEar | 0.000 | -0.025 | -0.618 | 0.567 | NS |
| geno1C1C2:orgJoint | 0.000 | -0.226 | -1.055 | 0.604 | NS |
| geno1C1C3:orgJoint | 0.000 | -0.550 | -1.199 | 0.099 | NS |
| geno1C2C2:orgJoint | 0.000 | 0.206 | -0.514 | 0.927 | NS |
| geno1C2C3:orgJoint | 0.000 | 0.750 | -1.304 | 2.803 | NS |
| geno1C3C3:orgJoint | 0.000 | 0.152 | -0.441 | 0.745 | NS |
| geno1C1C2:orgSkin | 0.000 | -0.575 | -1.404 | 0.254 | NS |
| geno1C1C3:orgSkin | 0.000 | -0.138 | -0.787 | 0.510 | NS |
| geno1C2C2:orgSkin | 0.000 | -0.086 | -0.807 | 0.635 | NS |
| geno1C2C3:orgSkin | 0.000 | 0.296 | -1.758 | 2.349 | NS |
| **geno1C3C3:orgSkin** | **0.000** | **-0.635** | **-1.228** | **-0.042** | **P<0.05** |
| geno3C1Cy:orgEar:sexM | 0.000 | -0.458 | -1.626 | 0.710 | NS |
| geno3CyCy:orgEar:sexM | 0.000 | -0.239 | -1.316 | 0.838 | NS |
| **geno3C1Cy:orgJoint:sexM** | **0.000** | **-1.445** | **-2.613** | **-0.278** | **P<0.05** |
| geno3CyCy:orgJoint:sexM | 0.000 | -0.821 | -1.898 | 0.256 | NS |
| geno3C1Cy:orgSkin:sexM | 0.000 | -0.661 | -1.829 | 0.507 | NS |
| geno3CyCy:orgSkin:sexM | 0.000 | 0.022 | -1.055 | 1.098 | NS |
| geno4C3Cz:orgEar:sexM | 0.000 | -0.564 | -1.818 | 0.689 | NS |
| geno4CzCz:orgEar:sexM | 0.000 | 0.315 | -0.715 | 1.345 | NS |
| geno4C3Cz:orgJoint:sexM | 0.000 | -0.659 | -1.913 | 0.594 | NS |
| geno4CzCz:orgJoint:sexM | 0.000 | 0.234 | -0.796 | 1.264 | NS |
| geno4C3Cz:orgSkin:sexM | 0.000 | -0.845 | -2.099 | 0.409 | NS |
| geno4CzCz:orgSkin:sexM | 0.000 | 0.271 | -0.759 | 1.301 | NS |
| geno6:orgEar:sexM | 0.000 | -0.091 | -0.633 | 0.451 | NS |
| geno6:orgJoint:sexM | 0.000 | -0.332 | -0.874 | 0.210 | NS |
| geno6:orgSkin:sexM | 0.000 | 0.064 | -0.478 | 0.606 | NS |
| geno5:orgEar:sexM | 0.000 | -0.103 | -0.737 | 0.531 | NS |
| geno5:orgJoint:sexM | 0.000 | 0.161 | -0.474 | 0.795 | NS |
| geno5:orgSkin:sexM | 0.000 | -0.300 | -0.935 | 0.334 | NS |
| geno7:orgEar:sexM | 0.000 | 0.196 | -0.323 | 0.714 | NS |
| geno7:orgJoint:sexM | 0.000 | 0.217 | -0.302 | 0.735 | NS |
| geno7:orgSkin:sexM | 0.000 | 0.189 | -0.329 | 0.708 | NS |
| geno2C2Cx:orgEar:sexM | 0.000 | 0.849 | -1.028 | 2.727 | NS |
| geno2CxCx:orgEar:sexM | 0.000 | 0.023 | -1.346 | 1.392 | NS |
| geno2C2Cx:orgJoint:sexM | 0.000 | 0.056 | -1.822 | 1.934 | NS |
| geno2CxCx:orgJoint:sexM | 0.000 | 0.352 | -1.017 | 1.721 | NS |
| geno2C2Cx:orgSkin:sexM | 0.000 | 0.239 | -1.638 | 2.117 | NS |
| geno2CxCx:orgSkin:sexM | 0.000 | -0.427 | -1.796 | 0.942 | NS |
| geno1C1C2:orgEar:sexM | 0.000 | 0.493 | -1.169 | 2.156 | NS |
| geno1C1C3:orgEar:sexM | 0.000 | -0.911 | -2.213 | 0.392 | NS |
| geno1C2C2:orgEar:sexM | 0.000 | -0.374 | -1.877 | 1.129 | NS |
| geno1C3C3:orgEar:sexM | 0.000 | -0.297 | -1.488 | 0.895 | NS |
| geno1C1C2:orgJoint:sexM | 0.000 | -1.219 | -2.882 | 0.443 | NS |
| **geno1C1C3:orgJoint:sexM** | **0.000** | **-1.540** | **-2.843** | **-0.237** | **P<0.05** |
| geno1C2C2:orgJoint:sexM | 0.000 | -1.076 | -2.579 | 0.427 | NS |
| geno1C3C3:orgJoint:sexM | 0.000 | -0.733 | -1.925 | 0.458 | NS |
| geno1C1C2:orgSkin:sexM | 0.000 | 0.237 | -1.426 | 1.899 | NS |
| geno1C1C3:orgSkin:sexM | 0.000 | -1.102 | -2.404 | 0.201 | NS |
| geno1C2C2:orgSkin:sexM | 0.000 | 0.104 | -1.399 | 1.607 | NS |
| geno1C3C3:orgSkin:sexM | 0.000 | -0.182 | -1.374 | 1.009 | NS |

**Section 8 – Materials and methods of the *Borrelia*-specific IgG antibody response**

At 35 days post-infection, a blood sample was collected from each animal (from the saphenous vein or the retro orbital sinus) to determine its infection status using ELISA. The SERION® ELISA classic *Borrelia burgdorferi* IgG/IgM immunoassay plates (Ruwag, Germany) were used to detect *Borrelia*-specific IgG antibodies following the manufacturer’s instructions. The wells were incubated with serum samples diluted to 1:100 in PBS for 45 min at room temperature. Each well was rinsed three times with 0.1% PBS-Tween before and after adding the secondary antibody for 45 min at room temperature. The secondary antibody was goat anti-*Mus musculus* IgG conjugated to horseradish peroxidase (Promega, Switzerland) and diluted 1:5000 in PBS. A volume of 100 μl of TMB product was added to each well. The absorbance was measured at 652 nm every 2 minutes for a total duration of 60 minutes. The strength of the *Borrelia*-IgG antibody response was calculated as the integral of the absorbance versus time curve over the total duration of 60 minutes of absorbance measurements. Positive controls were serum samples from *Mus musculus* mice that had been experimentally infected with *B. afzelii* isolate NE4049 via tick bite. Negative controls were serum samples from *M. musculus* mice that had never been exposed to ticks.

**Section 8 – Statistical analysis of the *Borrelia*-specific IgG antibody response**

Infection with *B. afzelii* induces a strong *Borrelia*-specific antibody response ^19^. To test whether our ELISA assay could discriminate between uninfected bank voles and *B. afzelii*-infected bank voles, the *Borrelia*-specific IgG antibody response was compared between these two groups with a t-test. This analysis was only done for the Swiss infection experiment, which had 16 uninfected individuals and 34 infected individuals. This analysis was not done for the Finnish infection experiment, because all 50 individuals were infected with *B. afzelii*.

To test whether the TLR2 genotype and sex influenced the *Borrelia*-specific IgG antibody response, we analysed the subset of 84 bank voles that were infected with *B. afzelii*. We applied the same model selection analysis approach that we had applied for the other response variables. The antibody response against *B. afzelii* was log10-transformed to normalize the residuals. This response variable was modelled using linear models with normal errors. The fixed factors were experiment, sex, and the eight different ways to code TLR2 genotype. Each vole occurred only once in the analysis so it was not necessary to include bank vole ID as a random effect.

**Section 8 – Results of the *Borrelia*-specific IgG antibody response**

As shown in previous studies ^20^, the goat anti-*Mus musculus* IgG secondary antibody was effective at binding the IgG antibodies of *M. glareolus*. The bank voles infected with *B. afzelii* had a *Borrelia*-specific IgG antibody response that was 5.2 times higher than that of the uninfected bank voles and this difference was highly significant (t = -11.978, df = 33.593, p-value < 0.001)*.* This result shows that the ELISA was highly effective for distinguishing between *B. afzelii*-infected and uninfected bank voles.

For the model selection analysis of the number of engorged nymphs per bank vole, the top 3 models had 99.997% of the support; the remaining 26 models had 0.003% of the support (**Table S15**). The support for individual factors was as follows: experiment (99.997%), sex (37.5%), and TLR2 genotype (<0.003% for 24 models). The model-averaged parameter estimates found that the bank voles in the Finnish experiment had a significantly higher antibody titre than the bank voles in the Swiss experiment (**Figure S3**; **Table S16**).


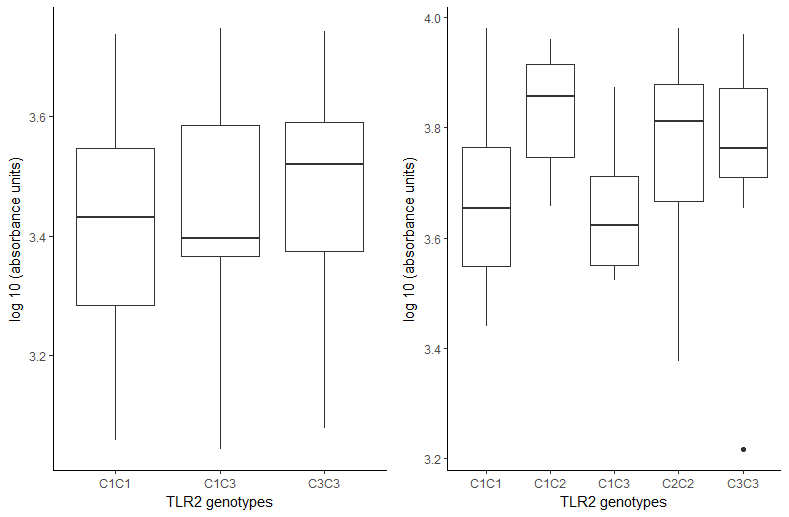


**Figure S3.** The TLR2 genotype of the bank vole has no effect on the *Borrelia*-specific IgG antibody response. The left and right panels refer to the Swiss infection experiment (n = 34 bank voles) and the Finnish infection experiment (n = 50), respectively. The *Borrelia*-specific IgG antibody response was measured using commercially available Lyme borreliosis ELISA plates. Absorbance values were integrated over the 60 minutes of the ELISA assay and log10-transformed to improve their fit to the normal distribution.

**Table S15.** Model selection table is shown for the linear models of antibody titres against *B. afzelii* (Ab.titre). The analysis was done on the subset of 84 bank voles infected with *B. afzelii*. Of the 29 models, the top 3 models have 99.997% of the support. The fixed factors are experiment (E), sex (S), and TLR2 genotype. TLR2 genotype was modelled in 8 different ways (geno1, geno2, geno3, geno4, geno5, geno6, geno7, and geno8). For each model, the model structure, degrees of freedom (df), log likelihood, corrected AIC value (AICc), difference in AICc from the top model (Delta), and the support (Weight) are shown.

| Model ID | Model Structure | df | logLik | AICc | delta | weight |
| --- | --- | --- | --- | --- | --- | --- |
| model003 | Ab.titre~E | 3 | 24.871 | -43.442 | 0.000 | 62.525 |
| model002 | Ab.titre~E+S | 4 | 25.032 | -41.558 | 1.883 | 24.383 |
| model001 | Ab.titre~E+S+E:S | 5 | 25.542 | -40.314 | 3.128 | 13.089 |
| model010 | Ab.titre~geno2 | 4 | 15.243 | -21.979 | 21.462 | 0.001 |
| model009 | Ab.titre~geno2+S | 5 | 15.377 | -19.985 | 23.457 | 0.001 |
| model022 | Ab.titre~geno5 | 3 | 12.888 | -19.477 | 23.965 | 0.000 |
| model021 | Ab.titre~geno5+S | 4 | 13.074 | -17.641 | 25.800 | 0.000 |
| model008 | Ab.titre~geno2+S+geno2:S | 7 | 16.530 | -17.586 | 25.856 | 0.000 |
| model019 | Ab.titre~C1+C2+C3 | 4 | 13.031 | -17.555 | 25.887 | 0.000 |
| model020 | Ab.titre~geno5+S+geno5:S | 5 | 13.713 | -16.657 | 26.784 | 0.000 |
| model007 | Ab.titre~geno1 | 7 | 15.911 | -16.349 | 27.093 | 0.000 |
| model018 | Ab.titre~C1+C2+C3+S | 5 | 13.200 | -15.631 | 27.810 | 0.000 |
| model006 | Ab.titre~geno1+S | 8 | 16.033 | -14.145 | 29.297 | 0.000 |
| model017 | Ab.titre~C1+C2+C3+S+C1:S+C2:S+C3:S | 7 | 14.321 | -13.168 | 30.274 | 0.000 |
| model025 | Ab.titre~geno6 | 3 | 7.910 | -9.519 | 33.922 | 0.000 |
| model029 | Ab.titre~1 | 2 | 6.209 | -8.271 | 35.171 | 0.000 |
| model028 | Ab.titre~geno7 | 3 | 7.047 | -7.793 | 35.649 | 0.000 |
| model013 | Ab.titre~geno3 | 4 | 8.125 | -7.744 | 35.698 | 0.000 |
| model024 | Ab.titre~geno6+S | 4 | 7.918 | -7.330 | 36.112 | 0.000 |
| model005 | Ab.titre~geno1+S+geno1:S | 12 | 17.751 | -7.108 | 36.334 | 0.000 |
| model016 | Ab.titre~geno4 | 4 | 7.422 | -6.338 | 37.104 | 0.000 |
| model004 | Ab.titre~S | 3 | 6.218 | -6.135 | 37.306 | 0.000 |
| model027 | Ab.titre~geno7+S | 4 | 7.083 | -5.660 | 37.782 | 0.000 |
| model023 | Ab.titre~geno6+S+geno6:S | 5 | 8.206 | -5.642 | 37.800 | 0.000 |
| model012 | Ab.titre~geno3+S | 5 | 8.132 | -5.495 | 37.947 | 0.000 |
| model015 | Ab.titre~geno4+S | 5 | 7.443 | -4.117 | 39.325 | 0.000 |
| model026 | Ab.titre~geno7+S+geno7:S | 5 | 7.232 | -3.695 | 39.746 | 0.000 |
| model011 | Ab.titre~geno3+S+geno3:S | 7 | 8.657 | -1.839 | 41.602 | 0.000 |
| model014 | Ab.titre~geno4+S+geno4:S | 7 | 8.153 | -0.832 | 42.610 | 0.000 |

**Table S16.** Model-averaged parameter estimates are shown for the linear models of antibody titres against *B. afzelii*. The model selection table contains 29 models. The fixed factors are experiment (expt), sex (sex), and TLR2 genotype. TLR2 genotype was modeled in 8 different ways (geno1, geno2, geno3, geno4, geno5, geno6, geno7, and geno8). For each of the 38 parameter estimates, mean1, mean2, the lower limit (LL) and upper limit (UL) of the 95% confidence interval (CI) for mean2, and the statistical significance (p < 0.05) are shown. Mean1 is averaged over all the models that contain that parameter in the model selection table. Mean2 is averaged over a subset of models that contain that parameter.

| Parameter | Mean1 | Mean2 | 95% LL | 95% UL | Signif |
| --- | --- | --- | --- | --- | --- |
| **Intercept** | **3.457** | **3.457** | **3.387** | **3.528** | **P<0.05** |
| **exptFin** | **0.280** | **0.280** | **0.189** | **0.371** | **P<0.05** |
| sexM | -0.002 | -0.006 | -0.113 | 0.102 | NS |
| exptFin:sexM | -0.011 | -0.081 | -0.243 | 0.082 | NS |
| geno2C2Cx | 0.000 | 0.073 | -0.136 | 0.281 | NS |
| **geno2CxCx** | **0.000** | **-0.196** | **-0.347** | **-0.046** | **P<0.05** |
| **geno5** | **0.000** | **-0.124** | **-0.201** | **-0.047** | **P<0.05** |
| geno2C2Cx:sexM | 0.000 | 0.257 | -0.116 | 0.630 | NS |
| geno2CxCx:sexM | 0.000 | 0.174 | -0.097 | 0.446 | NS |
| C1 | 0.000 | -0.018 | -0.083 | 0.047 | NS |
| **C2** | **0.000** | **0.113** | **0.039** | **0.187** | **P<0.05** |
| C3 | 0.000 | NA | NA | NA | NA |
| geno5:sexM | 0.000 | 0.072 | -0.057 | 0.202 | NS |
| **geno1C1C2** | **0.000** | **0.284** | **0.113** | **0.456** | **P<0.05** |
| geno1C1C3 | 0.000 | 0.001 | -0.133 | 0.136 | NS |
| **geno1C2C2** | **0.000** | **0.210** | **0.059** | **0.362** | **P<0.05** |
| geno1C2C3 | 0.000 | 0.371 | -0.053 | 0.795 | NS |
| geno1C3C3 | 0.000 | 0.057 | -0.066 | 0.180 | NS |
| C1:sexM | 0.000 | 0.056 | -0.067 | 0.178 | NS |
| C2:sexM | 0.000 | -0.051 | -0.191 | 0.090 | NS |
| C3:sexM | 0.000 | NA | NA | NA | NA |
| geno6 | 0.000 | 0.057 | -0.007 | 0.121 | NS |
| geno7 | 0.000 | 0.035 | -0.025 | 0.095 | NS |
| geno3C1Cy | 0.000 | 0.092 | -0.042 | 0.225 | NS |
| geno3CyCy | 0.000 | 0.116 | -0.007 | 0.240 | NS |
| geno1C1C2:sexM | 0.000 | 0.044 | -0.303 | 0.390 | NS |
| geno1C1C3:sexM | 0.000 | 0.039 | -0.233 | 0.310 | NS |
| geno1C2C2:sexM | 0.000 | -0.203 | -0.516 | 0.110 | NS |
| geno1C2C3:sexM | 0.000 | NA | NA | NA | NA |
| geno1C3C3:sexM | 0.000 | -0.091 | -0.339 | 0.158 | NS |
| geno4C3Cz | 0.000 | -0.039 | -0.187 | 0.109 | NS |
| geno4CzCz | 0.000 | 0.056 | -0.063 | 0.174 | NS |
| geno6:sexM | 0.000 | -0.045 | -0.164 | 0.075 | NS |
| geno7:sexM | 0.000 | 0.031 | -0.084 | 0.146 | NS |
| geno3C1Cy:sexM | 0.000 | 0.033 | -0.232 | 0.297 | NS |
| geno3CyCy:sexM | 0.000 | -0.078 | -0.322 | 0.166 | NS |
| geno4C3Cz:sexM | 0.000 | 0.163 | -0.121 | 0.447 | NS |
| geno4CzCz:sexM | 0.000 | 0.053 | -0.180 | 0.287 | NS |

References

1 Pérez, D., Kneubuhler, Y., Rais, O. & Gern, L. Seasonality of *Ixodes ricinus* ticks on vegetation and on rodents and *Borrelia burgdorferi* sensu lato genospecies diversity in two Lyme borreliosis-endemic areas in Switzerland. *Vector-Borne Zoonot.* **12**, 633-644 (2012).

2 Tschirren, B. *Borrelia burgdorferi* sensu lato infection pressure shapes innate immune gene evolution in natural rodent populations across Europe. *Biology Letters* **11** (2015).

3 Cayol, C. *et al.* *Borrelia afzelii* alters reproductive success in a rodent host. *P. Roy. Soc. B-Biol. Sci.* **285** (2018).

4 Kallio, E. R. *et al.* Cyclic hantavirus epidemics in humans - Predicted by rodent host dynamics. *Epidemics* **1**, 101-107 (2009).

5 Tschirren, B. *et al.* Contrasting patterns of diversity and population differentiation at the innate immunity gene toll-like receptor 2 (TLR2) in two sympatric rodent species. *Evolution* **66**, 720-731 (2012).

6 Stephens, M., Smith, N. J. & Donnelly, P. A new statistical method for haplotype reconstruction from population data. *Am. J. Hum. Genet.* **68**, 978-989 (2001).

7 Flot, J. F. SEQPHASE: a web tool for interconverting phase input/output files and fasta sequence alignments. *Molecular Ecology Resources* **10**, 162-166 (2010).

8 Clement, M., Posada, D. & Crandall, K. A. TCS: a computer program to estimate gene genealogies. *Mol. Ecol.* **9**, 1657-1659 (2000).

9 Belli, A., Sarr, A., Rais, O., Rego, R. O. M. & Voordouw, M. J. Ticks infected via co-feeding transmission can transmit Lyme borreliosis to vertebrate hosts. *Scientific Reports* **7**, 5006 (2017).

10 Jacquet, M., Durand, J., Rais, O. & Voordouw, M. J. Cross-reactive acquired immunity influences transmission success of the Lyme disease pathogen, *Borrelia afzelii*. *Infection Genetics and Evolution* **36**, 131-140 (2015).

11 Tonetti, N., Voordouw, M. J., Durand, J., Monnier, S. & Gern, L. Genetic variation in transmission success of the Lyme borreliosis pathogen *Borrelia afzelii*. *Ticks Tick Borne Dis.* **6**, 334-343 (2015).

12 Genné, D. *et al.* Competition between strains of *Borrelia afzelii* inside the rodent host and the tick vector. *P. Roy. Soc. B-Biol. Sci.* **285** (2018).

13 Jacquet, M., Margos, G., Fingerle, V. & Voordouw, M. J. Comparison of the lifetime host-to-tick transmission between two strains of the Lyme disease pathogen *Borrelia afzelii*. *Parasit. Vectors* **9** (2016).

14 Heylen, D. J. A. *et al.* Inefficient co-feeding transmission of *Borrelia afzelii* in two common European songbirds. *Scientific Reports* **7** (2017).

15 Schwaiger, M., Peter, O. & Cassinotti, P. Routine diagnosis of *Borrelia burgdorferi* (sensu lato) infections using a real-time PCR assay. *Clin. Microbiol. Infec.* **7**, 461-469 (2001).

16 Civitello, D. J. & Rohr, J. R. Disentangling the effects of exposure and susceptibility on transmission of the zoonotic parasite *Schistosoma mansoni*. *J. Anim. Ecol.* **83**, 1379-1386 (2014).

17 Tschirren, B. *et al.* Polymorphisms at the innate immune receptor TLR2 are associated with *Borrelia* infection in a wild rodent population. *P. Roy. Soc. B-Biol. Sci.* **280**, 20130364 (2013).

18 Kurtenbach, K. *et al.* Fundamental processes in the evolutionary ecology of Lyme borreliosis. *Nat. Rev. Microbiol.* **4**, 660-669 (2006).

19 Tracy, K. E. & Baumgarth, N. *Borrelia burgdorferi* manipulates innate and adaptive immunity to establish persistence in rodent reservoir hosts. *Frontiers in Immunology* **8** (2017).

20 Kurtenbach, K. *et al.* Differential immune responses to *Borrelia burgdorferi* in European wild rodent species influence spirochete transmission to *Ixodes ricinus* L (Acari, Ixodidae). *Infect. Immun.* **62**, 5344-5352 (1994).
